# Theoretical studies of modulation instability, Fermi–Pasta–Ulam recurrence and pattern formation in an ultra-silicon-rich-nitride Bragg grating

**DOI:** 10.1515/nanoph-2025-0073

**Published:** 2025-05-22

**Authors:** Amdad Chowdury, Benjamin J. Eggleton, Dawn T.H. Tan

**Affiliations:** 233793 Photonics Devices and Systems Group, Singapore University of Technology and Design, 8 Somapah Rd., Singapore 487372, Singapore; School of Physics, Institute of Photonics and Optical Science, The University of Sydney, Sydney, NSW 2006, Australia; Institute of Microelectronics, Agency for Science, Technology, and Research (A*STAR), 2 Fusionopolis Way, Singapore 138634, Singapore

**Keywords:** on-chip Bragg gratings, nonlinear optics, modulation instability, solitons, integrated photonics

## Abstract

Ultra-silicon-rich nitride Bragg gratings provide a powerful platform for precise light manipulation in photonic chips. Their exceptionally high nonlinearity and strong grating-induced dispersion near the stop-band edges significantly reduce the power and length required for chip-scale light–matter interactions. Using computational methods, we theoretically investigate modulational instability, Fermi–Pasta–Ulam recurrence, and pattern formation in this platform within the framework of the Akhmediev breather. We assess their experimental feasibility and show that this platform can generate a high-quality pulse train at the output. We demonstrate that modulational instability can be triggered in the gratings as short as 1–2 mm, leading to Akhmediev breather formation. By analyzing the full dispersion profile, we identify pump wavelengths that generate new frequencies and show that the grating also can produce a comb-like discrete spectrum. Furthermore, we reveal that even with high loss, parametric amplification at the grating output is possible, highlighting its potential as a nonlinear platform for frequency comb generation, wavelength-multiplexed data transmission, and high-precision pulse processing.

## 1 Introduction

Instability drives some of the most captivating and dynamic phenomena in nature, from the formation of weather systems to the birth of galaxies. In fluids, small instabilities can develop into turbulent flows, shaping ocean currents and atmospheric patterns [1], [2]. In optics, modulation instability (MI) – a complex nonlinear phenomenon transforms steady light waves into pulse trains, which are crucial for understanding laser dynamics and fiber optic communications [3], [4]. Even in biological systems, instabilities underpin pattern formation, from zebra stripes to neural activity [5], [6]. Studying the growth and evolution of these small perturbations reveals fundamental processes that govern the complexity and diversity of natural systems [7].

MI has been observed across various physical systems, including optics [8], fluid dynamics [9], [10], [11], plasma physics [12], biophysics [5], and self-organizing pattern formations [13]. In optics, MI occurs when a continuous wave (CW) travels through a nonlinear medium, such as an optical fiber or waveguide. Through the interaction of nonlinearity and dispersion, small perturbations in amplitude or phase grow exponentially, causing the CW to break into a pulse train which in the frequency domain, creates a comb-like spectrum. This process can lead to soliton generation and the formation of complex, dynamic patterns, making MI essential to understanding light behavior in nonlinear media. Beyond optical communications, MI serves as a model for exploring the emergence of chaos and turbulence in other nonlinear systems.

The study of MI has a rich history, uncovering numerous novel phenomena and insights [14], [15]. Decades of focused research have produced an extensive body of literature spanning both theoretical and experimental work. The first exploration of MI in nonlinear media was conducted by Soviet physicists in the early 1960s [16], [17], [18], with its first theoretical prediction in optical fibers reported by Hasegawa [19], [20], [21]. Experimental verification was achieved in the early 1980s by Tai and collaborators [4], highlighting the critical role of anomalous dispersion in driving MI. These foundational works demonstrated that MI is strongly enhanced under anomalous dispersion, establishing its significance in fiber optics. Subsequent research on MI has led to major discoveries, including the Benjamin–Feir instability [22], Fermi–Pasta–Ulam (FPU) recurrence [23], rogue waves [24], and supercontinuum generation [25], [26], each illustrating MI’s role in revealing the intricate dynamics of nonlinear wave systems.

While much of the early research focused on optical fibers, advancements in dispersion engineering have brought new platforms into focus. Fiber Bragg gratings (FBGs), first demonstrated in 1978 [27], marked a pivotal development in controlling light propagation. It was Winful’s discovery that gratings exhibit much higher dispersion than glass [28] that spurred interest in exploring nonlinear phenomena, including MI, in FBGs [29], [30], [31], [32].

Advancements in high-quality integrated photonic devices have enabled researchers to study MI in novel geometries and materials [33], [34]. Nonlinear waveguides fabricated on silicon, silicon nitride, and Hydex platforms provide compact, well-controlled environments for investigating MI dynamics [35]. Recent progress in microfabrication has further shifted the focus toward chip-based Bragg gratings, which offer substantial benefits, such as reduced size, lower cost, and enhanced integration [34], [36], [37], [38]. Primary studies of MI dynamics in on-chip Bragg gratings have uncovered new insights in the areas of integrated photonics and expanded the possibility of their range of potential applications [33], [39], [40].

Building on these advances in integrated photonic platforms, a theoretical understanding of MI remains crucial for designing and optimizing such systems. The nonlinear Schrödinger equation (NLSE) is widely regarded as the key equation for analyzing MI in fiber systems [3], [4], [41]. It offers a robust framework to describe the evolution of slowly varying wave packets in a nonlinear medium, where small perturbations can grow exponentially to form highly localized structures [3], [42], [43], [44], [45].

However, pulse dynamics in fiber Bragg gratings differ significantly from those in standard fibers. First, dispersion in fiber Bragg grating is nearly six orders of magnitude higher than in conventional fibers [46], and second, pulse propagation is much slower in them due to multiple reflections at the grating interfaces, fundamentally altering the pulse dynamics [30]. To address these unique characteristics, Winful derived two coupled mode equations for the forward and backward propagating fields within the fiber Bragg grating structures [28]. Sterke and colleagues further demonstrated that for MI studies, these coupled mode equations can be approximated by a unidirectional NLSE when field intensity is low, while the full coupled mode equations are essential for high-intensity input optical fields [47], [48], [49].

Building on these findings, similar behavior has been observed in chip-based Bragg gratings, which exhibit strong dispersion and enhanced nonlinearity [34], [50], [51]. Higher-order dispersion terms play a critical role in the nonlinear interactions within these gratings, and the generalized nonlinear Schrödinger equation (GNLSE), which includes both higher-order dispersion and nonlinear effects [3], serves as a fundamental tool for accurately modeling light propagation in such systems [34], [52].

Recent advancements in on-chip ultra-silicon-rich nitride (USRN) waveguides further extend these capabilities, offering a promising photonic platform that enables strong light–matter interactions at an unprecedented shorter than millimeter-scale lengths [34], [53], [54]. A chip-scale Bragg grating on USRN material can achieve a nonlinear refractive index 1,000 times greater and group velocity dispersion (GVD) approximately 6 orders of magnitude higher than conventional fibers [34], [55]. Studies using GNLSE already have explored phenomena in USRN Bragg gratings, such as Bragg soliton fission and pulse compression [55], quartic Bragg soliton generation [56], spectral broadening [57], [58], and picosecond pulse generation [40]. However, research on MI dynamics and other crucial nonlinear phenomena associated with it on this platform remains largely unexplored.

In this work, we employ the GNLSE to investigate MI [15] and several nonlinear phenomena that emerge from it, including Akhmediev breather (AB) dynamics [59], FPU recurrence [23], and pattern formation [60], in USRN Bragg gratings. Although similar phenomena have been extensively studied and observed in large-scale nonlinear platforms such as fibers, laser cavities, plasma, and fluid dynamics [7], in this work, we demonstrate their observation at a significantly smaller scale and with reduced power requirements. We begin by studying noise-induced MI across the grating’s full dispersion range, showing the generation of new wavelengths beyond the pump. This is followed by a detailed analysis of MI in the grating using linear stability analysis. Using the inverse scattering transform (IST), we confirm the existence of an AB-type solution within the grating, providing a deeper understanding of its nonlinear behavior.

Utilizing the AB solution, we investigate two additional nonlinear phenomena: FPU recurrence and pattern formation. This demonstrates that USRN Bragg gratings support a diverse range of nonlinear interactions on a single, integrated platform. The AB solution not only sets the initial conditions for examining MI but also links the physical parameters of the grating through its internal mathematical structure, offering a comprehensive explanation of observed behaviors. Using this framework, we show that in the chaotic regimes, the complete dispersion profile facilitates MI, leading to AB formation and a continuous spectrum. Conversely, in deterministic regimes, only a portion of the dispersion profile promotes AB development, resulting in a discrete spectrum. Furthermore, this solution elucidates the distinct nature of FPU recurrence and pattern formation in systems characterized by extreme nonlinearity and dispersion, markedly different from conventional nonlinear systems.

Furthermore, we investigate the experimental feasibility of MI, FPU recurrence, and pattern formation in USRN Bragg gratings, focusing on the power and length requirements necessary for their observation. Unlike fiber Bragg gratings and other MI-based systems, USRN Bragg gratings exhibit a more intricate dispersion profile, resulting in complex MI band structures that span a wide range of spectral regions. We analyze how the MI bandwidth and spectral features evolve with increasing power and examine the conditions under which these nonlinear phenomena can emerge across a broad power range, from several watts down to tens of milliwatts. Furthermore, we explore how the position of seeding frequencies within the MI bands influences the system’s dynamical behavior, leading to standard MI, higher-order MI dynamics, FPU recurrence, and pattern formation.

## 2 Device platform: the USRN-Bragg grating

**Figure 1:**
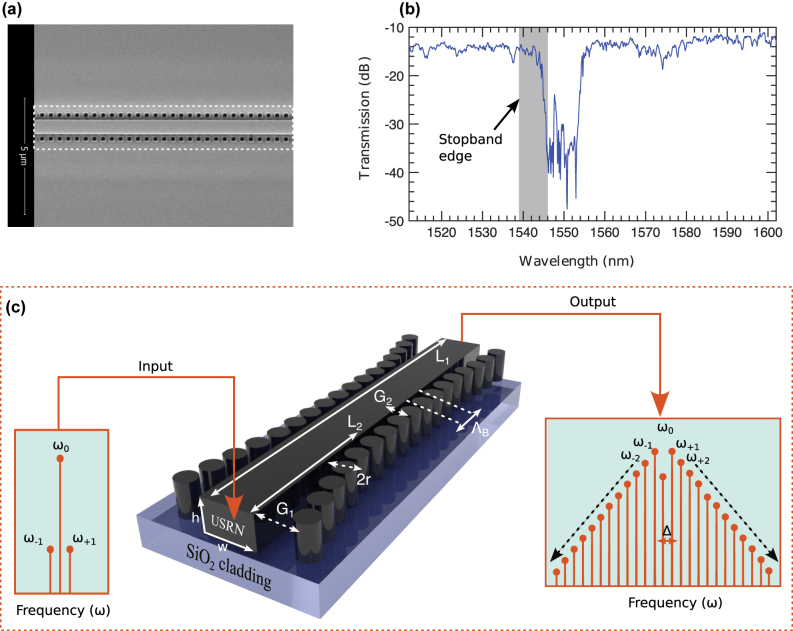
Device description: (a) the SEM image and (b) experimentally measured transmission band of the grating. (c) 3D-schematic of the Bragg grating with cladding-modulated gratings structures. Its length *L*_1_, height *h* and width *w*. Other grating parameters are: pitch Λ_*B*_, gaps *G*_1_ and *G*_2_ and pillar radius *r*. The input and output schematically show how an incident optical field is modified after traveling through the USRN Bragg gratings.

This work presents a purely theoretical and numerical investigation of nonlinear dynamics in a USRN Bragg grating. Although the analysis is entirely based on simulations, it is grounded in the actual physical characteristics of a previously fabricated and characterized device. To provide clarity and context, Figure 1 includes the full device configuration, a scanning electron microscope (SEM) image of the fabricated grating, and the experimentally measured transmission spectrum. These elements are included solely to illustrate that the parameters used in the theoretical modeling – such as the dispersion profile and transmission characteristics – are derived from a real device. No new experimental measurements are reported in this study. The goal is to explore additional continuous-wave-based nonlinear phenomena that such a grating can support, extending the understanding of its theoretical potential that can be harnessed in experiments and applications.

### 2.1 Tailorable refractive index

Silicon-rich nitride has recently gained attention as a promising material for integrated photonic platforms [50]. Its refractive index can be tuned between 2.1 and 3.1 by tailoring the silicon content, allowing optimized designs for photonic devices like waveguides, resonators, and modulators [51]. This tunability is essential for meeting specific optical requirements, such as phase matching in nonlinear processes or minimizing losses.

The Kramers–Kronig relation provides a foundation for silicon-rich nitride-based material design, showing that the refractive index increases as the bandgap decreases, while absorption drops with a larger bandgap [61], [62]. These principles, related to Miller’s rule, indicate that nonlinear polarizability (*χ* ^3^) scales with the refractive index *n* [63]. Carefully balancing bandgap, refractive index, and absorption is crucial for selecting silicon-rich nitride materials for nonlinear applications. Minimizing two-photon absorption at telecom wavelengths (1,550 nm), while maintaining a high nonlinear coefficient, is a key objective for effective material design.

### 2.2 Compatibility with CMOS fabrication

Silicon has advanced as a waveguide material and serves many applications [50], but it faces challenges as a nonlinear material. Its indirect bandgap (1.1 eV at room temperature) reduces two-photon absorption at 1,550 nm, but it can still occur at high intensities, converting optical energy into heat and limiting nonlinear effects like four-wave mixing and supercontinuum generation [64]. Additionally, free carriers cause extra losses through absorption and affect dispersion, degrading the performance of nonlinear optical devices [65], [66], [67].

To address these challenges, CMOS-compatible silicon nitride has emerged as a promising alternative. Beyond mitigating the drawbacks of silicon, silicon nitride offers excellent compatibility with CMOS processes, enabling scalable integration with application-specific integrated circuits. This compatibility has driven advancements in key technologies, such as on-chip frequency combs, high-density optical communications, and quantum entanglement systems. Furthermore, the ability to tune the silicon-to-nitrogen ratio in silicon nitride provides precise control over its nonlinear and dispersion properties, paving the way for the exploration of novel nonlinear phenomena at the chip scale [29], [55], [68].

Building on these developments, the USRN Bragg grating represents a next-generation platform that capitalizes on the benefits of silicon nitride while further enhancing nonlinear interactions. The USRN Bragg grating is constructed on a silicon-rich nitride platform with a silicon-to-nitrogen ratio of 7:3, resulting in a high silicon content that yields a large nonlinear refractive index of 2.9 × 10^−17^ m^2^/W and a linear refractive index of 3.1 at 1,550 nm. Fabricated using inductively coupled chemical vapor deposition at 250 °C, this low deposition temperature makes the platform ideal for CMOS backend processing [34].

While the USRN platform avoids two-photon absorption at 1,550 nm, it does exhibit three-photon absorption at the Urbach tail [69]. However, three-photon absorption impacts nonlinear efficiency far less than two-photon absorption. The high nonlinear parameter of USRN enhances nonlinear interactions, optimizing conversion efficiency in processes like four-wave mixing and optical parametric gain, which is particularly advantageous for achieving high conversion at low input powers [3].

### 2.3 Grating design

Figure 1 illustrates the design of a USRN Bragg grating. In Figure 1(a), an SEM image shows the 6 mm grating structure, while Figure 1(b) presents its transmission band. The shaded region near the stop-band edge, spanning wavelengths from 1,539 nm to 1,546 nm, demonstrates strong dispersion due to multiple reflections at the band edge [30]. A 3D schematic of the grating is provided in Figure 1(c). The design achieves high-value dispersion by incorporating cladding-modulated Bragg gratings on both sides of the central waveguide *L*_1_, which measures 6 mm × 600 nm × 300 nm. Precise dispersion engineering is enabled by adjusting parameters such as the pillar radius *r*, gap *G*_1_ (distance from the waveguide), and grating pitch Λ_*B*_. To ensure smooth optical field interaction with the waveguide, the input and output gratings are apodized, resulting in a gradual effective index change and minimizing abrupt field transitions. A comprehensive description of the grating design and its parameters is provided in [55]. The detailed description of material properties, fabrication conditions, and geometric parameters provided in this section ensures the completeness and reproducibility of the device configuration used in this study.

## 3 Theroretical framework

*Modellig pulse dynamics with GNLSE*: Modelling pulse dynamics with GNLSE: In our modeling, we employed GNLSE to capture the evolution of the forward-propagating pulse within the Bragg grating structure. While this approach does not explicitly include a spectral filter or account for backward-propagating waves within the stopband (and as a result, nonzero spectral components may appear within the stopband in our simulations), it effectively describes the key nonlinear dynamics in the transmission region and provides insight into the nonlinear phenomena that arise as a result of the complex interplay between nonlinearity, dispersion and slow light elements within the grating.

*The Akhmediev Breather (AB) Solution:* In 1984, Hasegawa used the NLSE to study MI and pulse train generation in fiber systems through numerical simulations [19]. A year later, Akhmediev and colleagues derived the exact NLSE solution for CW modulation, now known as the Akhmediev breather or AB solution, which provides an accurate model for MI dynamics [70]. The AB solution has garnered significant attention in both optics and hydrodynamics due to its ability to explain MI and rogue wave formation in these fields [71], [72]. In fiber optics, the AB describes how small perturbations on a CW grow into periodic pulse trains [59] or develop into optical rogue waves [73]. In hydrodynamics, it models the sudden appearance of high-amplitude oceanic rogue waves with destructive potential [9], [11]. Over the decades, extensive research has expanded our understanding of MI and the AB solution, resulting in a vast body of literature [24], [74]. However, the dynamics of MI and other nonlinear phenomena within the framework of the AB solution in chip-scale integrated Bragg gratings remain largely unexplored. This study aims to address this gap, offering new insights into the rich nonlinear behavior in integrated photonic systems.

In the NLSE, dispersion originates from the fiber material itself, while in the Bragg grating, dispersion arises from the periodic effective index modulation, functioning similarly to the dispersive term in the NLSE. In a grating when the frequency of the propagating field closely aligns with the frequency at the stop-band edge and intensity stays below a threshold, coupled mode equations can approximate NLSE behavior, as shown in several studies [30], [55], [75]. In this work, the cladding-modulated Bragg grating operates within these optical frequency and intensity constraints, making NLSE suitable for analysis. However, due to higher-order dispersion in the grating, we apply the GNLSE to study the MI and AB dynamics [3], [55]:

(1) $$\frac{\partial \psi }{\partial z}=-\frac{\alpha \;\psi }{2}+i\;\;\overset{4}{\underset{n=2}{\sum }}\frac{i^{n}}{n!}\;\beta_{n}\;\;\frac{\partial^{n}\psi }{\partial t^{n}}+i\;\gamma \;\;\left(|\psi {|}^{2}\psi \right)$$

When *α* = *β*_3_ = *β*_4_ = 0 and *β*_2_ = −1, this is the integrable NLSE and it has exact soliton and AB solutions [59], [76]. Here *z* and *t* are the evolution and the transverse variable. *ψ*(*z*, *t*) is the pulse envelop. *β*_2_ < 0 is the GVD parameter measured in the unit of ps^2^mm^−1^ and *β*_3_ and *β*_4_ are the third-order dispersion (TOD) and fourth-order dispersion (FOD) parameter with the unit of ps^3^mm^−1^ and ps^4^mm^−1^. *γ* is the nonlinear parameter quantified in *W* ^−1^. mm^−1^. |*ψ*|^2^ can be considered the instantaneous power *P*_0_. The close form AB solution of NLSE can be given by [70]:

(2) $$\psi \left(z,t\right)=\sqrt{P_{0}}\;\times \left(\frac{\left(1-4a\right)\cosh\left(bz\right)+ib\sinh\left(bz\right)+\sqrt{2a}\cos\left(\omega_{mod}t\right)}{\sqrt{2a}\cos\left(\omega_{mod}t\right)-\cosh\left(bz\right)}\right)$$

This solution provides us with numerous initial conditions matching with experimental setup to study MI effectively in a range of nonlinear systems. Here, *P*_0_ is the pump power, and the key parameter *ω*_mod_ represents the perturbation frequency required to excite an AB. This frequency perturbs the CW, leading it to grow exponentially and evolve into the AB described by this solution. The modulation frequency *ω*_mod_ is determined by the system’s nonlinear and dispersion parameters, with *a* and *b* defined in terms of *ω*_mod_. The parameter *b*, also known as the growth rate, is dictated by the parameter *a* and indicates how fast an AB develops. The maximum frequency capable of exciting a breather is denoted by *ω*_c_. These parameters are related by the following equations:

(3) $$\begin{array}{cc} \omega_{c} & =\sqrt{\left(4\;\gamma \;P_{0}\right)/|\beta_{2}|}\\ a & =\frac{1}{2}\left[1-{\left(\omega_{mod}/\omega_{c}\right)}^{2}\right],\;\;\omega_{c}>\omega_{mod}>0\\ b & =\sqrt{8a\left(1-2a\right)},\;\;\;0<a<1/2 \end{array}$$

When *a* = 1/4, the AB’s growth rate reaches its maximum, with *b* = 1 and a modulation frequency of $\omega_{mod}=\omega_{\text{c}}/\sqrt{2}$ . To maintain the validity of the AB solution, *ω*_mod_ must lie within the range *ω*_*c*_ > *ω*_mod_ > 0. The period of the AB is defined as *T*_mod_ = 2*π*/*ω*_mod_, where *ω*_mod_ serves as the angular frequency characterizing the periodicity of the AB.

*Generating continuous spectrum:* To generate a continuous spectrum in the grating numerically, the initial condition is given by:

(4) $$\psi \left(z=0,t\right)=\sqrt{P_{0}}\left[1+a\left(t\right)+i\;b\left(t\right)\right]$$

where *P*_0_ is the pump power. This initial condition is not directly related to Eq. (2); however, an AB-type solution still emerges in the evolution field under this setup, even within a chaotic wave environment [77]. Here, *a*(*t*) and *b*(*t*) are two uncorrelated, real, random functions that vary within a small range around zero. This expression triggers MI within the grating, amplifying only those frequencies within the gain band. Due to the chaotic, continuous nature of the initial condition, many frequency components fall within the AB’s gain band. Consequently, not only the frequency with the highest gain will be amplified, but neighboring frequencies will also grow in intensity [77], [78]. However, the frequencies with the highest gain will reach peak intensity and grow faster than the others.

*Generating discrete spectrum:* To generate a discrete spectrum in the grating, we derive our initial condition from Eq. (2). At *z* = 0, however, Eq. (2) remains complex, making it unsuitable as an initial condition to numerically excite an AB, and its complexity also poses challenges for experimental implementation [77]. Instead, we use the alternative, real expression [8], [79], [80]:

(5) $$\psi \left(z=0,t\right)=\sqrt{P_{0}}\left[1+\epsilon_{mod}\cos\left(\omega_{mod}t\right)\right]$$

where *ϵ*_mod_ and *ω*_mod_ are the modulation amplitude and frequency respectively. With this initial condition, MI allows the grating to develop an AB with a discrete spectral profile. Due to waveguide losses, power in the system decreases, so *ω*_*c*_ in Eq. (3) is modified to *ω*_*c*_ = *ω*_*c*_ exp(−*αz*/2), significantly impacting the AB’s growth rate [3]. Note that the growth rate expression here aligns with that in [3].

*MI study using linear stability analysis:* To analyze MI systematically, we apply linear stability analysis, yielding the growth rate expression [81], [82]:

(6) $$\begin{array}{ll} g\left(\omega \right) & =I\left\{\beta_{2}^{2}\omega^{2}\left(\omega^{2}+\omega_{c}^{2}\right)\right.\\ & \;{\left.+\frac{\beta_{4}}{144}\left[12\beta_{2}\omega^{4}\left(2\omega^{2}+\omega_{c}^{2}\right)+\beta_{4}\omega^{8}\right]\right\}}^{1/2} \end{array}$$

Setting *β*_4_ = 0 simplifies Eq. (6), reducing it to the standard NLSE growth rate without contributions from higher-order dispersion terms. While this linear stability analysis excludes the effects of TOD on MI, subsequent numerical methods have shown that TOD introduces subtle features that are not captured by the linear approach. Despite this limitation, the simplified expression in Eq. (6) provides a fast and efficient method for analyzing MI dynamics in a controlled manner.

*The IST Analysis:* To confirm the presence of an AB in the grating, we perform an IST analysis to identify its unique spectral signature, which may arise during CW propagation. The process starts with the standard integrable NLSE expressed in its Lax pair form – two linear equations representing the spatial and temporal components, associated with eigenvalues and eigenfunctions [83], [84]. This formulation, also known as the Zakharov–Shabat scattering problem [76], allows us to extract eigenvalues by using the solution profile at any point along the grating as the potential. These eigenvalues constitute the IST spectral signature, which is distinct for each solution type. By comparing the IST spectral portrait obtained from the grating’s evolution field with known solutions, we determine whether the observed solution corresponds to an AB or a soliton-type structure. Further details on this process are provided in the Methods section.

## 4 Results

### 4.1 Noise-induced MI in the grating: continuous output spectrum

**Figure 2:**
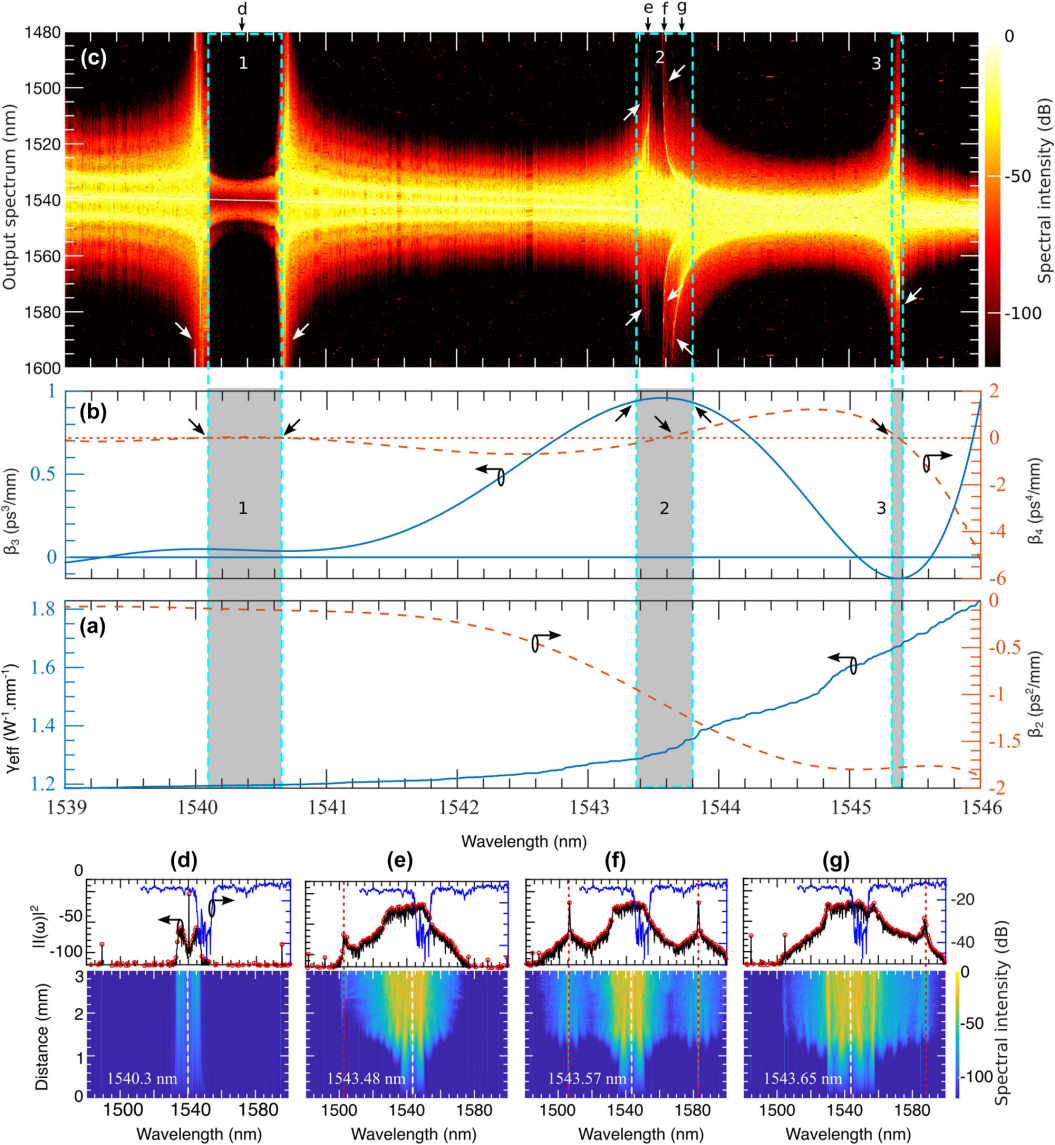
Noise-induced MI driven by the device’s dispersion and nonlinear parameters: (a) and (b) present the experimentally measured effective nonlinear parameter (*γ*) and dispersion parameters (*β*_2_, *β*_3_, *β*_4_) across the wavelength range of 1,539 nm–1,546 nm which are used in the numerical simulation. Using this range as the pump, (c) shows how noise-induced MI generates a new spectrum. The shaded regions in (a) and (b) correspond to specific parameter values that define three distinct regions in (c): region **1**, where no new wavelengths are generated, and regions **2** and **3**, where new spectral components are formed. The bottom panels, (d), (e), (f), and (g), illustrate the spectral evolution at four selected wavelengths indicated by black arrows in (c). In these panels, dashed white lines mark the pump wavelength, red dotted lines indicate the newly generated spectral components and the transmission bands are shown in blue lines.

At the edge of the stopband, the grating’s dispersion profile becomes highly complex, with higher-order dispersion terms playing a significant role. This complexity makes predicting the grating’s response to variations in the input wavelength challenging. Figure 2(a) presents the GVD and nonlinear parameter values, while Figure 2(b) displays the TOD and FOD parameters. In the wavelength range of 1,539–1,546 nm, there are 4,999 pump wavelengths spaced approximately 176 MHz apart. Each pump is characterized by specific values for the nonlinear parameter *γ* and dispersion parameters *β*_2_, *β*_3_, and *β*_4_. These parameters, combined with the initial condition given in Eq. (4), were used to solve Eq. (1). The results are shown in Figure 2(c), where the *x*-axis represents the pump wavelengths, and the *y*-axis displays the output spectrum.

The grating’s resonance response shows distinct features in regions labeled 1, 2, and 3 in Figure 2(c), corresponding to the dispersion and nonlinear parameter values within the shaded areas of Figure 2(a) and (b). In region 1, the narrow bandwidth and negligible frequency excitation result from the minimal values of *β*_2_ and *β*_4_ in the corresponding shaded area. The growth rate *g*(*ω*) from Eq. (6) indicates that when *β*_2_ = *β*_4_ ≈ 0, the MI gain bandwidth is highly restricted, resulting in almost no amplification of new frequency components and a vanishing growth rate. Although nonlinear effects *γ* and TOD may induce weak self-phase modulation and MI [3], their contribution remains insignificant.

We observe an abrupt widening of the output spectrum at both edges of the region 1, around 1,540 nm and 1,540.6 nm, driven by variations in *β*_4_. These broadenings are indicated by the white arrows in Figure 2(c) and the corresponding *β*_4_ values indicated with black arrows in Figure 2(b). The sign of *β*_4_ plays a crucial role: *β*_4_ > 0 but ≪ 1 enhances MI, resulting in spectral broadening, while *β*_4_ < 0 or *β*_4_ ≫ 1 suppresses MI, confining spectral components near the pump [82], [85]. Around 1,540 nm, for a narrow range of pump wavelengths with small positive values of *β*_4_, generate multiple MI gain bands, producing new frequencies farther from the pump and briefly widening the output spectrum. Within the 1,540.10–1,540.58 nm range, nonlinear interactions are significantly reduced because *β*_4_ has positive higher values, which also reduce the spectral bandwidth. In this range, the nonlinear parameter *γ* and dispersion parameters *β*_2_ and *β*_3_ remain nearly constant. Another widening of the spectrum is observed around 1,540.6 nm for a narrow range of pump wavelengths, as shown in region 1 of Figure 2(c). Here, the broad spectral components also arise due to *β*_4_ > 0 but *β*_4_ ≪ 1 values. It is important to note that for enhanced seeding and spectral amplification, FOD must remain positive, but with *β*_4_ ≪ 1 [82].

This behavior highlights FOD as a key control parameter for determining how the grating generates new wavelengths. This phenomenon is well-explained by the analytic gain expression in Eq. (6), a sixth-order polynomial in frequency *ω*. When *β*_4_ > 0, the condition *g*(*ω*)′ = 0 (where the prime denotes the first derivative with respect to *ω*) yields four MI gain bands symmetrically positioned on both sides of the pump [82], [85]. As the noisy CW pump evolves, frequencies within these gain bands experience strong phase-matching, amplifying spectral components away from the pump. In contrast, when *β*_4_ < 0, *g*(*ω*)′ = 0 produces only two symmetrical gain bands centered around the pump, without additional MI sub-bands. This narrows the gain spectrum and confines the instability region near the pump frequency, limiting the extent of spectral broadening.

To further examine the grating’s response within the narrow bandwidth region labeled 1 in Figure 2(c), we selected a pump wavelength of 1,540.3 nm, marked by the black arrow at *d*. The simulated result, shown in the bottom panel in Figure 2(d), reveals no spectral broadening along the grating. The developing MI experiences little to no growth for this range of wavelengths and no development of ABs in this region. In the upper panel of Figure 2(d), the black curve indicates minimal spectral change at the grating’s output. The blue curve above shows the grating’s transmission band, providing a comparison with the output spectrum.

The region 2 in Figure 2(c) displays intriguing spectral features corresponding to the shaded area 2 in Figure 2(a) and (b), covering the wavelength range from approximately 1,543.4 nm to 1,543.8 nm. Within this range, both symmetric and asymmetric spectral wings emerge, marked by arrows *e*, *f*, and *g*. For the asymmetric spectral broadening, pump wavelengths in this region exhibit positive TOD values, as shown by the peak of the blue curve in Figure 2(b) within the shaded area 2. These TOD values dominate over the effects of FOD, enhancing MI and amplifying the asymmetric spectral wings. In contrast, symmetric spectral broadening arises where the FOD curve crosses the zero line and *β*_4_ > 0. Small positive *β*_4_ values generate two symmetric resonance wings.

Alternatively, all downward black arrows in Figure 2(b) indicate the positions of small *β*_4_ values, which contribute to the symmetric broad spectrum in Figure 2(c), marked by downward white arrows. Similarly, all the upward black arrows in Figure 2(b) correspond to the positive TOD values responsible for the asymmetric spectrum in Figure 2(c), highlighted by upward white arrows. The symmetric and asymmetric spectral features are associated with pump wavelengths at 1,543.48 nm, 1,543.57 nm, and 1,543.65 nm. Their corresponding spectral evolutions are presented in Figure 2(e)–(g), respectively, in the bottom panel.

A notable feature in Figure 2(e) is the delayed emergence of the phase-matched wavelength, which becomes evident around *z* ≈ 2.5 mm, as indicated by the red dashed line. The high loss within the grating initially causes an exponential power reduction, suppressing MI and delaying the appearance of phase-matched frequencies. Additionally, at this wavelength, the TOD values correspond to small negative FOD values, which further suppress MI. As the evolution progresses, the exponential growth of MI eventually overcomes this suppression, resulting in a modest spectral gain at the grating’s output. This is reflected in the upper spectral wings along the black arrow *e* in Figure 2(c).

In contrast, Figure 2(f) and (g), corresponding to arrows *f* and *g* in Figure 2(c), show regular early MI development without delay. While linear stability analysis (Eq. (6)) predicts that *β*_3_ (TOD) does not directly contribute to MI development, this analysis reveals asymmetric spectral features driven by TOD. Further investigation is needed to understand how TOD influences MI in the presence of FOD.

In regions 1 and 3, where broader spectral output occurs for a narrow range of pump wavelengths, the spectral evolution resembles that shown in Figure 2(f) with a symmetric profile, though with varying spectral bandwidths. Additional details on the temporal and spectral evolution near these pump wavelengths are provided in the Supplementary material (see Figure 1).

**Figure 3:**
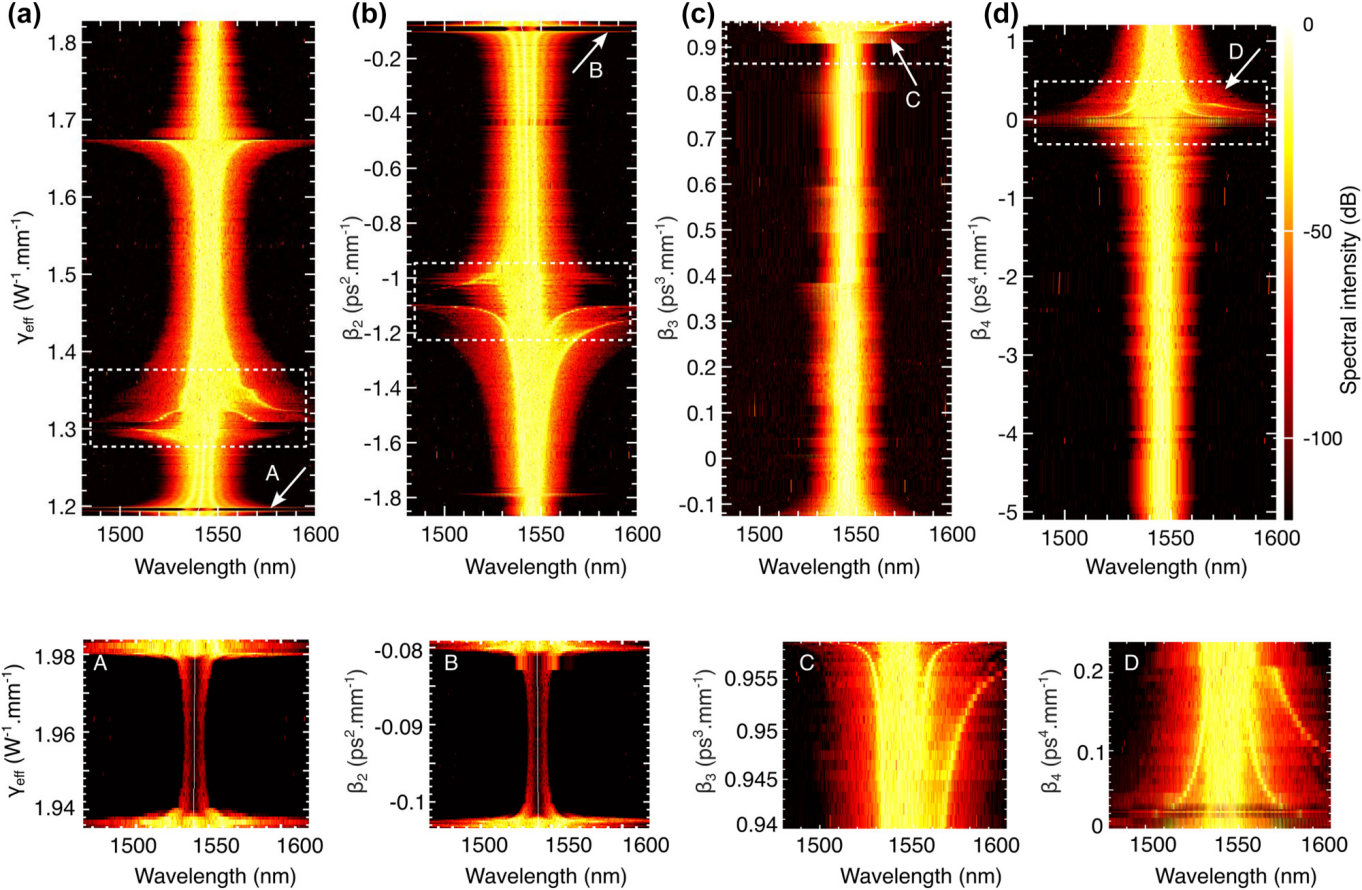
Individual contributions of nonlinear and dispersion parameters: (a) shows the contribution of the nonlinear parameter (*γ*), while (b), (c), and (d) illustrate the contributions of the dispersion parameters *β*_2_, *β*_3_, and *β*_4_, respectively. Key features are highlighted with white arrows, and enlarged views of these regions are presented in **A**, **B**, **C**, and **D** in the bottom panel.

While Figure 2(c) demonstrates the combined effects of nonlinear and dispersion parameters on the output spectrum, analyzing the individual contributions of these parameters to MI can provide valuable insights for optimizing grating performance through precise dispersion engineering. In Figure 3, we identify the ranges of *γ*, *β*_2_, *β*_3_, and *β*_4_ that drive the generation of new spectral components. Notably, the region 2 in Figure 2(c), characterized by multiple spectral wings, corresponds to rapidly changing sections of the dispersion and nonlinear parameter curves. In Figure 3(a)–(d), the nonlinear and dispersion values within the white boxes align with region 2 in Figure 2(a)–(c), revealing their role in producing multiple wide spectral regions.

The data presented in Figure 3 are derived from experimentally measured dispersion and nonlinearity parameters obtained across a dense set of discrete wavelengths in the 1,539–1,546 nm range (see Methods). For the purpose of numerical modeling and visualization, these curves were interpolated using a reduced number of wavelength points. Since the effective nonlinearity *γ* and second-order dispersion *β*_2_ exhibit nearly linear dependence over the measured range, their interpolated profiles appear smooth and monotonic in Figure 3(a) and (b). In contrast, the higher-order dispersion terms *β*_3_ and *β*_4_ exhibit strong nonlinear dependence on wavelength, with inflection points and curvature. Consequently, approximating these curves with fewer points leads to moderate point-to-point variations, resulting in the less monotonic trends observed in Figure 3(c) and (d).

The spectral shape and orientation within these white boxes vary significantly, as seen in Figure 2(c). However, further investigation is needed to uncover the intricate relationships among these parameters and how they shape the resulting spectral content.

Additionally, we observe a gap in region 1 of Figure 2(c), where nonlinear interactions are minimal. This gap reappears in Figure 3(a) and (b) when analyzing *γ* and *β*_2_, marked by white arrows *A* and *B*. These values correspond precisely with shaded region 1 in Figure 2(a), indicating that *γ* and *β*_2_ are the primary contributors to nonlinear interactions in this part of the grating. Conversely, the absence of similar gaps in Figure 3(c) and (d) suggests that *β*_3_ and *β*_4_ play a lesser role in these interactions. Enlarged views of key features are presented in the bottom panels *A*− *D*.

Furthermore, we observe a symmetric, wider spectral content at *γ* = 1.67 and *β*_2_ = −1.8 in Figure 3(a) and (b), respectively. These correspond to the points where the *β*_4_ curve intersects the zero line (red-dotted) in region 3 of Figure 2(b). These specific parameter values are directly responsible for generating the corresponding wide spectrum in Figure 2(c), highlighting the crucial interplay between these parameters in shaping the output spectral characteristics.

A notable general feature arises from the contribution of *β*_4_. In Figure 2(b), every instance where the *β*_4_ curve intersects the zero line results in a wider spectral regime in Figure 2(c), regardless of the behavior of other parameters (see all the downward black arrows). This underscores the pivotal role of *β*_4_ in determining the overall spectral behavior of the grating.

### 4.2 MI dynamics using linear stability analysis

**Figure 4:**
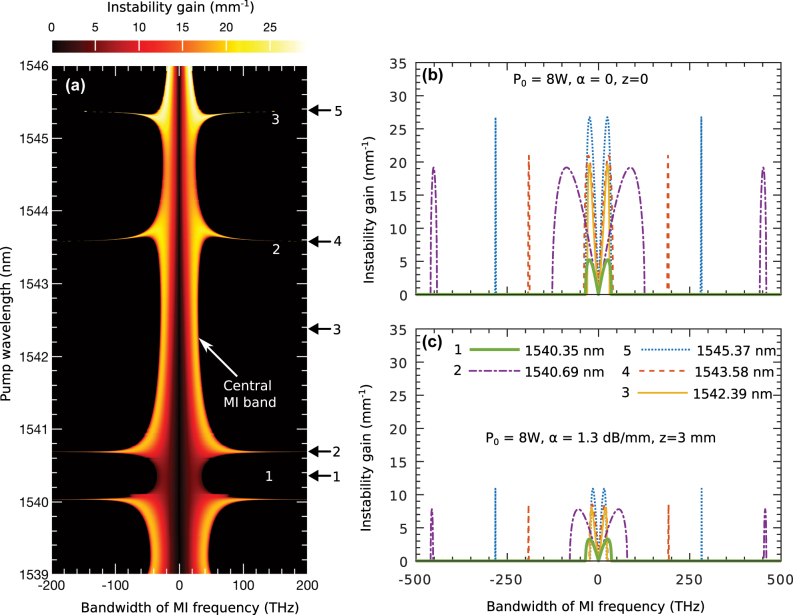
MI using linear stability analysis: (a) shows the output MI frequencies (*x*-axis) as a function of input pump wavelengths (*y*-axis) at a pump power of *P*_0_ = 8 W, without accounting for loss in Eq. (6). The pump wavelengths correspond to the nonlinear and dispersion values shown in Figure 2(a) and (b). (b) presents the output frequencies for five selected pump wavelengths. (c) shows the same five examples as in (b), but with loss included at the same power level.

MI in the grating can also be analyzed using linear stability analysis [3]. One may wonder: if MI can be studied analytically through linear stability, why use computationally expensive numerical methods? The answer lies in the limitations of linear stability analysis. The MI expression, Eq. (6), is derived without including the third-order dispersion (TOD) term *β*_3_. Using this expression, we generate the MI structure for the grating’s nonlinear and dispersion parameters, as shown in Figure 4(a). However, the numerical analysis incorporates all dispersion and nonlinear terms, producing the MI structure in Figure 2(c). Comparing Figure 4(a) with Figure 2(c), it is evident that several spectral wings, missing in the analytical result, appear in the numerical MI structure. These additional spectral components, which arise from TOD, are crucial for a comprehensive understanding of MI and can only be captured through numerical methods.

In Figure 4(a), the *y*-axis represents pump wavelengths, while the *x*-axis displays the corresponding MI frequency bandwidth. These pump wavelengths are associated with the nonlinear and dispersion parameters shown in Figure 2(a) and (b). For clarity, the analysis assumes zero loss and a pump power of *P*_0_ = 8 W. Regions 1, 2, and 3 in Figure 4(a) correspond to those in Figure 2(c). Note that Figure 4(a) highlights the MI frequencies responsible for initiating instabilities, in contrast to Figure 2(c), which presents the output spectrum.

To explore the impact of grating loss on MI dynamics, we analyze specific pump wavelengths marked by black arrows in Figure 4(a). Figure 4(b) shows the corresponding MI bandwidths without loss, with an expanded *x*-axis to display the full range of MI frequencies. In Figure 4(b), arrow 1 at 1,540.35 nm, located in a region of minimal nonlinear interaction, generates a narrow MI band with a lower growth rate, shown by the thick green curve. Pump wavelengths at arrows 2, 4, and 5 produce additional MI subbands (purple dot-dashed, brick-red dashed, and blue dotted lines) with varying bandwidths and growth rates, resulting in strong MI consistent with the wide spectral wings observed in Figure 2(c). Arrow 3, however, at 1,542.39 nm, does not generate MI subbands and shows a narrow MI bandwidth, represented by the solid yellow line.

When loss is introduced, the MI dynamics change significantly. Setting *P*_0_ = 8 W and a loss of *α* = 1.3 dB/mm, Figure 4(c) displays the MI structure at the end of the 3 mm grating. Loss reduces both the MI bandwidth and growth rate. As a result, narrower-band, lower-intensity spectral components dominate the output. Importantly, the location of the MI subbands remains unchanged despite the inclusion of loss.

**Figure 5:**
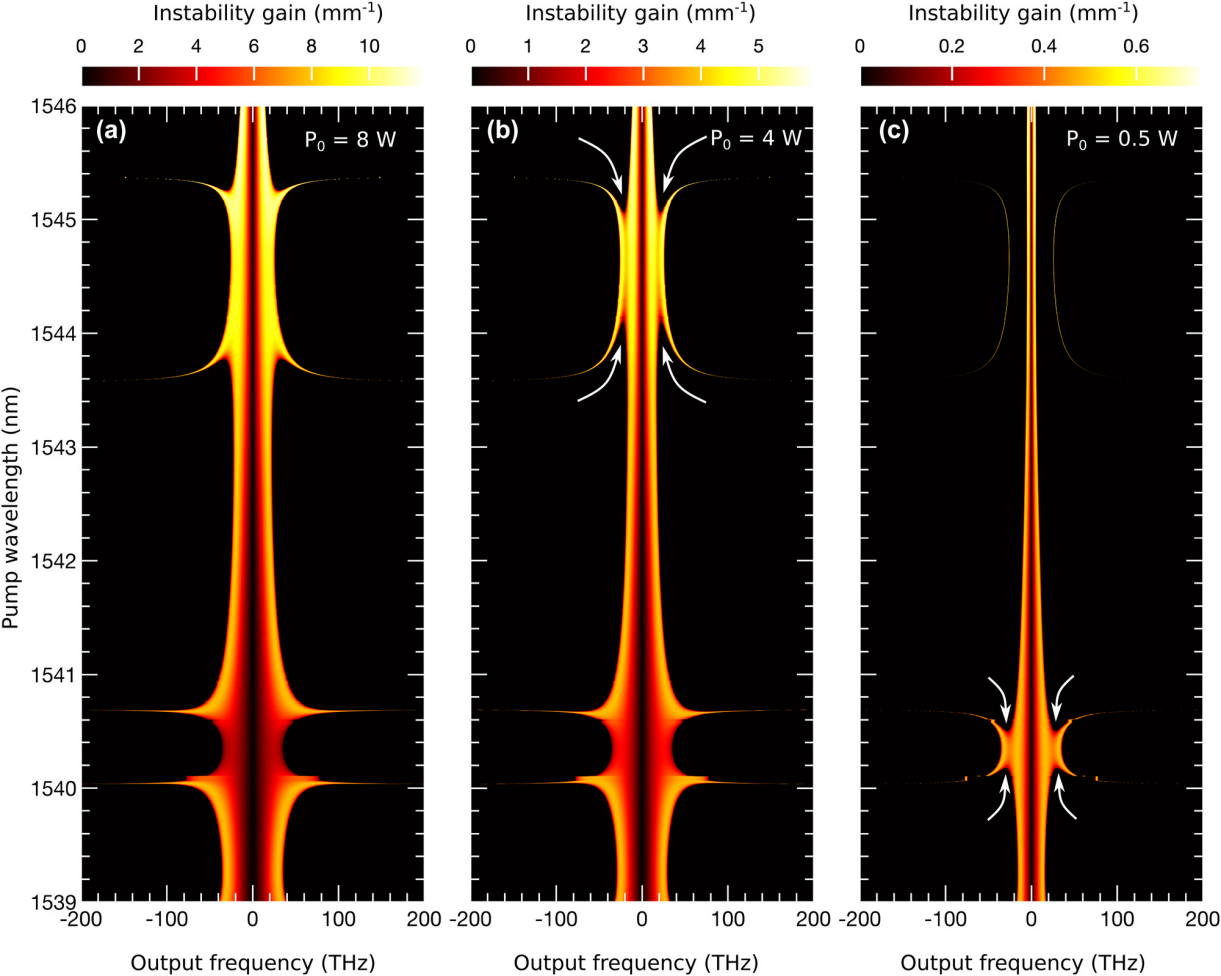
Power dependence of the grating’s MI: (a) shows the MI spectrum at *P*_0_ = 8 W with loss, corresponding to the example in Figure 4(c). (b) and (c) present the spectra at lower powers, *P*_0_ = 4 W and *P*_0_ = 500 mW, respectively, with loss included in all cases. The white arrows indicate the separation of the resonant MI sub-bands from the central MI band as the power decreases.

Figure 5 illustrates how the MI band structure varies with power in the presence of loss. Figure 5(a) corresponds to the MI bands at 8 W with waveguide loss, showing reduced bandwidth compared to Figure 4(a). As the power decreases to 4 W in Figure 5(b), the MI bandwidth narrows further, and distinct features emerge. Specifically, the resonant structure begins to separate from the central MI band, as marked by the curved white arrows. At lower power levels, such as 500 mW in Figure 5(c), the upper MI resonance region is nearly eliminated, leaving the lower resonance region still connected to the central MI band. Separation from the main MI band becomes more pronounced as power decreases further, with additional details provided in the Supplementary material (see Figure 3).

These power-dependent changes in the MI band structure are critical for understanding MI dynamics at the chip scale. On-chip gratings and waveguides often operate at power levels in the range of hundreds to tens of milliwatts. At these lower powers, the MI bandwidth becomes narrow, allowing only a limited number of MI frequencies to drive instability. However, the separation of MI subbands from the central band at lower powers enables phase-matched MI frequencies in the separated subbands, supporting strong MI even at milliwatt power levels.

### 4.3 Emergence of AB in the grating confirmed by inverse scattering transform (IST) analysis

The IST is a powerful and well-established method for deriving closed-form solutions to the NLSE [86], [87]. Beyond its original purpose, IST has been applied to solve practical problems in optical communications. In 1993, Hasegawa and colleagues proposed an efficient fiber communication system based on IST [88], and more recently, it has been used to encode, transmit, and process data by mitigating nonlinear channel cross-talk in optical fiber systems [89].

In nonlinear wave systems such as fibers, waveguides, and gratings, the evolution of CW fields often leads to the formation of localized structures, including solitons, ABs, and rogue waves [90]. Identifying these solutions within a chaotic wave field is challenging, but numerical techniques combined with statistical methods have been employed to address this [71], [91], [92]. Recently, IST has emerged as an effective tool for identifying and classifying localized nonlinear structures within chaotic wave fields [93], [94], [95]. Moreover, it has been applied to study how chaotic initial conditions in the NLSE evolve into solitons, ABs, and rogue wave solutions [96], [97].

While most IST analyses for communication and localized structure identification have been conducted in fiber systems, their application in on-chip data processing remains largely unexplored. Before adopting the AB model to explain MI and related nonlinear phenomena in these gratings, it is essential to confirm that they do emerge in this system. To achieve this, we employ the IST technique, as previously used to identify AB-type solutions in [94].

**Figure 6:**
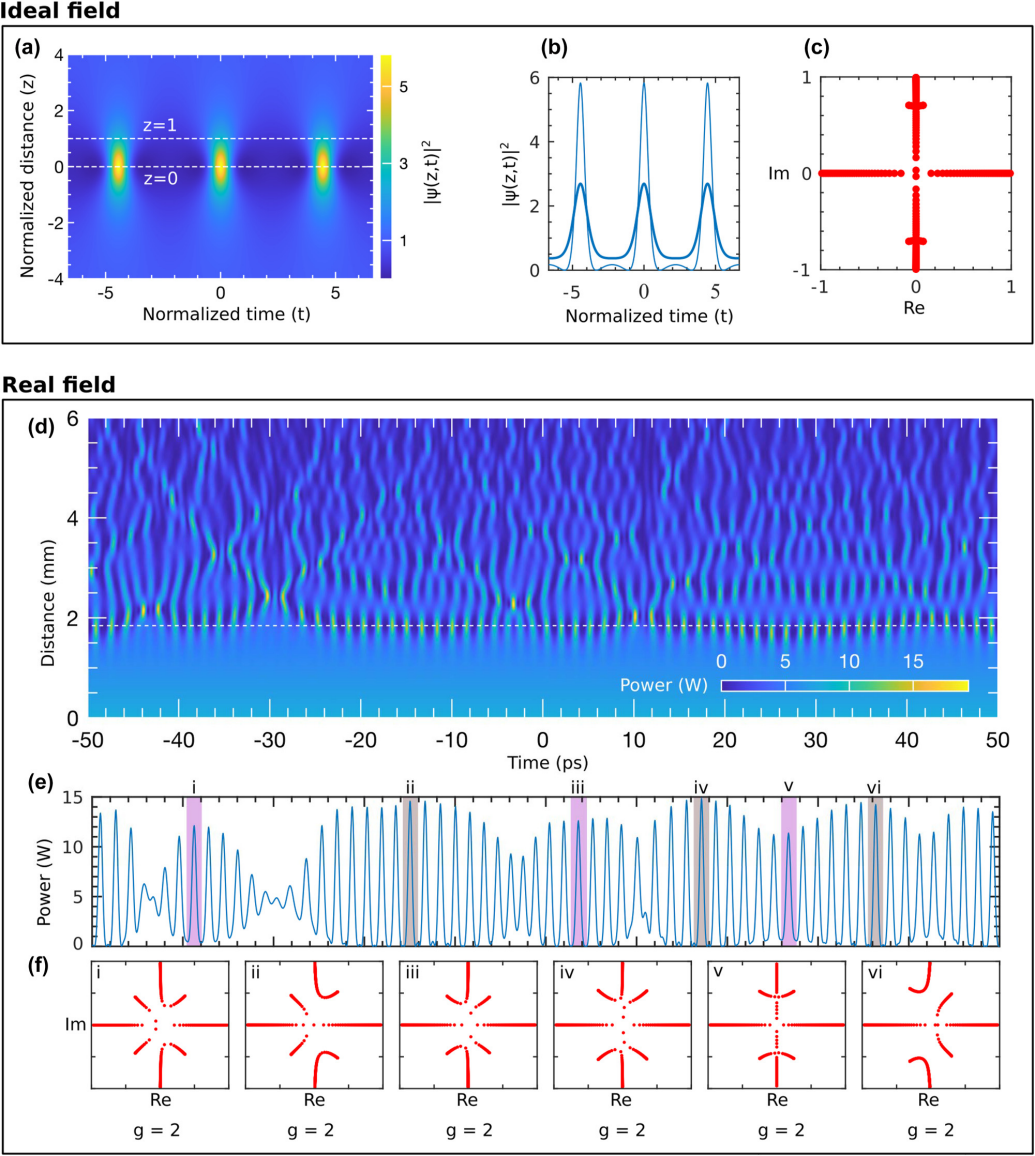
IST spectral analysis of the emergence of AB in the grating: [Ideal field] (a) shows the intensity profile of an ideal AB solution derived from the NLSE. Two intensity profiles, selected at *z* = 0 and *z* = 1 (indicated by white dashed lines), are presented in (b) as thin and thick blue curves, respectively. (c) displays their corresponding IST spectra. [Real field] (d) shows the evolution of a CW pump at 1,542 nm over a full 6 mm grating length. A temporal frame of intensity selected at *z* = 1.9 mm (marked by the white dashed line) is shown in (e). Six localized structures (*i − vi*) from the envelope are selected for IST spectral analysis, and their corresponding IST spectra are shown in (f) within the labeled boxes (*i − vi*). The genus (*g*) of each structure is indicated at the bottom. Note that (d) and (e) has the same *x*-axis.

The top panel of Figure 6 illustrates the IST analysis of an ideal periodic AB solution. Figure 6(a) shows the evolution of the AB from a dimensionless distance of −4 to 4, with three consecutive periods along the transverse dimension *t*. The solution is symmetric at both ends. For IST spectral analysis, intensity profiles at *z* = 0 and *z* = 1 are selected, shown in Figure 6(b) as thin and thick curves. The profile at *z* = 0 displays maximum intensity, which decreases significantly by *z* = 1. However, the IST spectra for both profiles, shown in Figure 6(c), remain identical, confirming that the IST spectra of an AB solution do not depend on the propagation distance [94], particularly near *z* = 0. Later, we confirm that this property also holds for ABs formed through CW propagation in the grating. Note, far from the AB’s peak intensity at *z* = 0, the IST spectrum resembles that of a plane wave [45].

The bottom panel of Figure 6 presents the IST analysis of a real optical field. Figure 6(d) shows the evolution of a CW in a 6 mm grating. Initially, strong nonlinear interactions and MI compress the CW, forming an AB-like wave profile around *z* ≈ 1.9. As the wave propagates, the grating loss parameter gradually reduces nonlinear interactions and MI, resulting in low-intensity localized structures at its output. With a loss of 1.3 dB/mm, the grating incurs a total loss of approximately 7.8 dB over its entire length, leaving only 17 % of the initial power at the output. Consequently, intense nonlinear interactions are primarily confined to the first ≈3mm , with significantly reduced intensity thereafter.

We extract the transverse profile at *z* ≈ 1.9, where the AB achieves maximum compression, as marked by the white dashed line in Figure 6(d). The wave envelope at this point is shown in Figure 6(e). To verify that the localized formations within the envelope correspond to AB solutions, six structures labeled *i* through *vi* are highlighted in different colors. Their corresponding IST spectra, or “IST spectral portraits,” are presented in the bottom panel.

To confirm these spectral portraits correspond to AB-type solutions, we analyze them using finite-gap theory [84]. According to this theory, IST spectral data consists of spectral bands and gaps, characterized by ‘genus-g’ [93], [94]. The genus *g* of an NLSE solution is related to the number of gaps as *g* = *N* − 1, where *N* is the number of spectral bands [45]. For instance, a plane wave has *g* = 0, a soliton has *g* = 1, and an AB solution has *g* = 2. The IST spectra of the six localized structures show *N* = 3 spectral bands, confirming their genus *g* = 2 and verifying that they are AB-type solutions.

It is noteworthy that the wave envelope in Figure 6(d) does not perfectly align with the AB’s maximum compression point due to the highly modulated chaotic wave field that disrupts the symmetry observed in regular AB solutions in Figure 6(b). Structures in the pink-shaded areas (*i*, *iii*, *v*) fall slightly before or after this point, while those in the gray-shaded areas (*ii*, *iv*, *vi*) align more closely. Despite this, all structures share the same AB-type IST spectral portrait, illustrating that the eigenvalues of the Zakharov–Shabat problem remain independent of propagation distance [93], [94], as also emphasized in the ideal IST analysis in the upper panel. We analyzed and described the IST spectrum of the entire envelope in the Supplemental material (see Figure 4), highlighting that most of the localized structure within the envelop displays characteristic AB behavior.

### 4.4 Exciting AB in the grating: discrete output spectrum

An AB at its maximum compression point exhibits a discrete spectral pattern [98]. To excite an AB solution and generate such a spectrum within the grating, we employed the initial condition $\psi \left(z=0,t\right)=\sqrt{P_{0}}\left[1+\epsilon_{mod}\cos\left(\omega_{mod}t\right)\right]$ (Eq. (5)) to solve the GNLSE. Experimentally, this setup corresponds to a modulated CW laser at the grating input, a standard configuration for AB studies in optical fibers, particularly at the telecom wavelength of 1,550 nm [80], [99]. Simulations utilized the full range of nonlinear and dispersion values across the wavelengths shown in Figure 2(a), with a power of *P*_0_ = 8 W and modulation amplitude *ϵ*_mod_ = 10^−4^.

**Figure 7:**
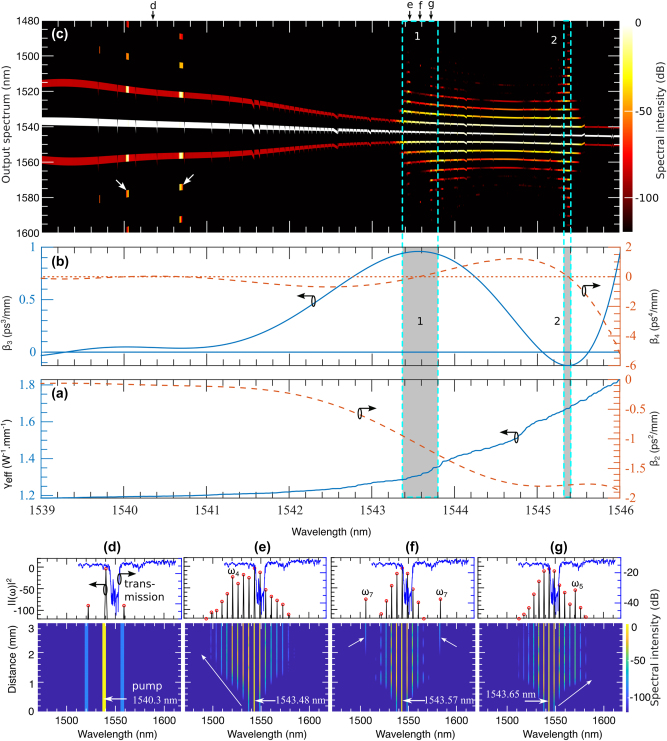
Generating ABs through CW wave modulation: (a) shows the nonlinear parameter (*γ*) and the group velocity dispersion (*β*_2_), while (b) presents the higher-order dispersion parameters (*β*_3_ and *β*_4_). (c) illustrates the output spectrum of ABs generated based on the nonlinear and dispersion values from (a) and (b). The shaded regions indicate phase-matched areas that result in new frequency generation, labeled as regions **1** and **2** in (c). The bottom panels, (d), (e), (f), and (g), depict the spectral evolution at selected pump wavelengths (highlighted in white text) indicated by their position by arrows in (c). A large portion of the grating’s dispersion profile, spanning from ≈ 1,539 nm to 1,543.4 nm, is identified as not supporting AB formation.

Figure 7(a) and (b) shows pump wavelengths along the *x*-axis, with each pump associated with unique nonlinear and dispersion values, while the *y*-axis in Figure 7(c) presents the output spectrum. Interestingly, the grating does not support AB excitation for a significant portion of the pump wavelengths. The central high-intensity white line in Figure 7(c) represents the output pump, while the two modulation sidebands remain visible but muted within the 1,539–1,543.30 nm range. AB formation begins only when the pump reaches 1,543.4 nm, producing new frequencies up to 1,545.6 nm.

This absence of AB excitation can be explained using the AB parameters in Eq. (2). The modulation frequency *ω*_mod_ relates to the parameter *a* as $2a=1-{\left(\omega_{mod}/\omega_{c}\right)}^{2}$ , where $\omega_{c}^{2}=4\gamma P_{0}/|\beta_{2}|$ . AB formation requires *ω*_*c*_ > *ω*_mod_ > 0, allowing MI to grow within the gain band defined by $b=\sqrt{8a\left(1-2a\right)}$ . However, in the grating, higher-order dispersion terms are significant. The MI gain expression for the standard AB solution, which includes only *β*_2_, does not fully apply. For AB excitation, the MI gain expression Eq. (6), incorporating *β*_4_, must hold. In the range 1,539–1,543.30 nm, large values of *γ*, *β*_2_, and *β*_4_ significantly narrow the MI gain band, pushing MI frequencies outside the band and preventing AB growth. Notably, *β*_4_ is largely negative in this range but briefly becomes positive near 1,540–1,540.6 nm, generating ABs and discrete spectra, as indicated by the white arrows in Figure 7(c).

The resonance frequencies or phase-matched new frequencies shown in Figure 7(c) align closely with Figure 2(c). Regions 1 and 2 in Figure 7(c) correspond to regions 2 and 3 in Figure 2(c). In Region 1, high loss reduces the visibility of resonance frequencies. Higher-order dispersion plays a critical role in generating discrete frequencies, with prominent spectral features near the stopband edge (1,543.4–1,545.6 nm) where *β*_4_ > 0. This demonstrates that the grating’s dispersion near the stopband edge is crucial for phase-matched spectral generation. We also examine the individual contributions of nonlinear (*γ*) and dispersion parameters (*β*_2_, *β*_3_, and *β*_4_) to the generation of discrete spectrum in the Supplementary material (see Figure 5).

Figure 7(d), representing a pump wavelength at 1,540.3 nm, shows no AB development. Here, modulation sidebands around the pump remain inactive, generating no new frequencies along the grating’s evolution. In contrast, Figure 7(e)–(g) demonstrates new frequency generation, highlighting the grating’s ability to amplify harmonic frequencies depending on phase-matching contributions from *β*_3_ or *β*_4_. Asymmetric amplification arises from *β*_3_, while symmetric amplification is driven by *β*_4_. These effects are marked by white arrows in the spectral profiles.

Note that the apparent difference in spectral resolution among Figure 7(d)–(g) arises from the dependence of the modulation frequency and time window on the second-order dispersion |*β*_2_|. A smaller |*β*_2_| results in a higher modulation frequency and thus a shorter time window *t*, which lowers the spectral resolution and causes the sidebands to appear broader. This effect is reflected in the initial spectral components shown in Figure 7(c). In contrast, larger |*β*_2_| values lead to a longer *t*, yielding higher spectral resolution and sharper, more distinct sideband structures. This trend is clearly observable in Figure 7(c), where the pump and the first two sidebands appear thicker at the beginning and progressively become narrower and more resolved along the dispersion profile.

In Figure 7(c), the central white line represents the output pump, while sidebands initially appear symmetrically around it. As pump wavelengths approach 1,546 nm, the sidebands converge. This behavior reflects the dispersion curves in Figure 7(a): lower dispersion values at shorter pump wavelengths support wideband MI, placing sidebands farther from the pump. As dispersion values increase, the MI band narrows, bringing sidebands closer to the pump and producing a converging spectral profile. Note that the black and white spikes appearing at various spectral positions, including along the central white pump line, are numerical artifacts and are not associated with any physical effects.

In the next sections, we explore FPU recurrence, pattern formation, and spectral enhancement in the grating within the AB framework, illustrating its versatility as a nonlinear platform.

### 4.5 FPU recurrence in the grating

The FPU recurrence is a fascinating nonlinear phenomenon with an equally intriguing history [100]. It describes how an active nonlinear system periodically returns to its initial state [23]. Since its discovery, this phenomenon has profoundly influenced nonlinear science, with applications ranging from wave dynamics to complex systems [101], [102], [103], [104]. The dynamics of the AB solution align closely with the behavior described by FPU recurrence [77], [104], [105]. In AB dynamics, a small modulation on a CW field causes exponential amplification of two sidebands. Through four-wave mixing, these sidebands generate new harmonic frequencies, drawing energy from the CW. This process continues until it reverses and returns to its initial state, illustrating the FPU recurrence. We show that the USRN Bragg grating, as a nonlinear platform, can also exhibit this cyclical process.

**Figure 8:**
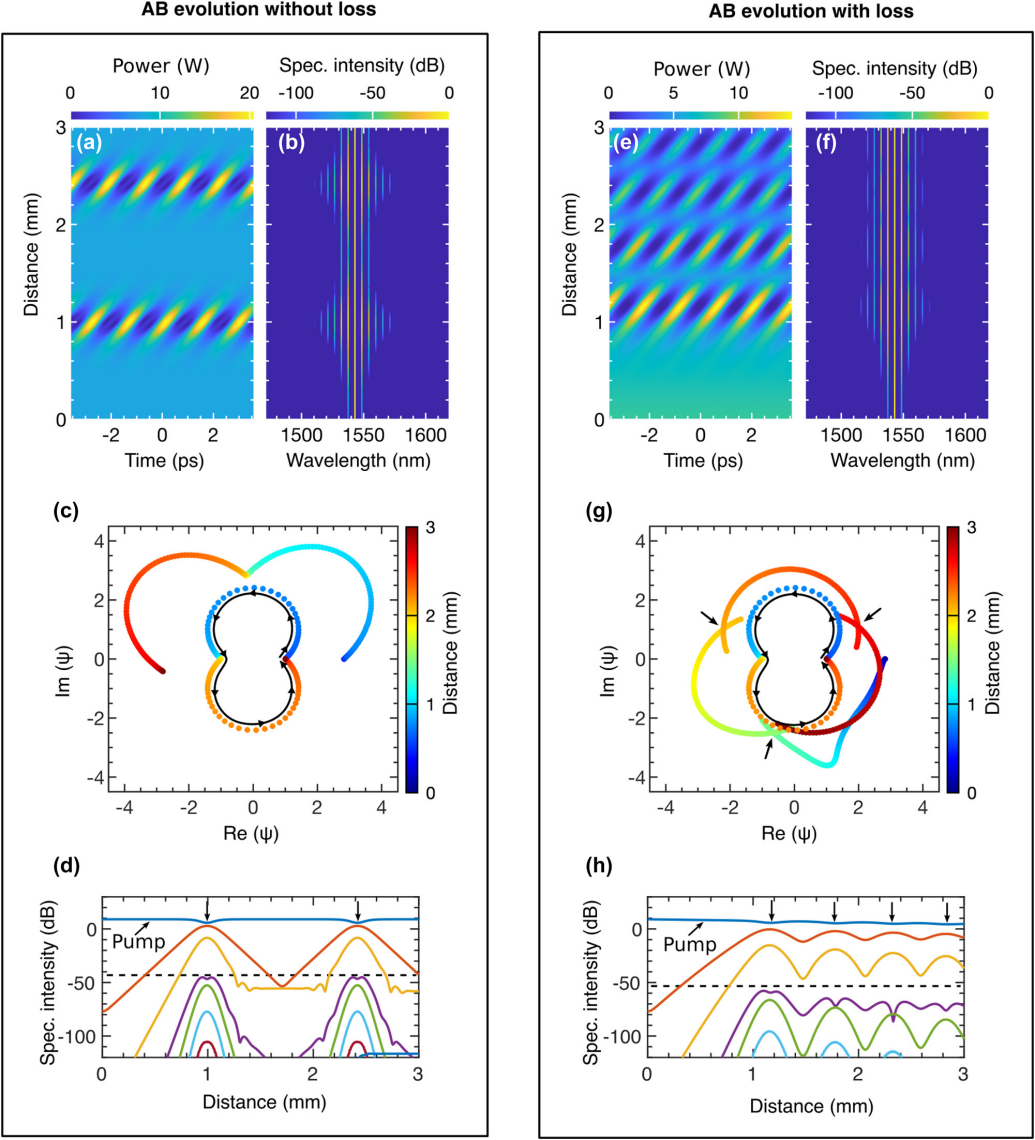
Observing FPU recurrence without and with waveguide loss: [Left box] (a) shows the temporal and (b) the spectral evolution of an AB at *λ*_0_ = 1,543.57 nm with no loss (*α* = 0). (c) depicts the temporal evolution of the AB’s amplitude on a complex plane, while (d) illustrates the energy exchange among spectral sidebands during evolution. [Right box] (e) and (f) show the temporal and spectral evolution, respectively, when the grating experiences a loss of 1.3 dB/mm. (g) demonstrates the effect of loss on the AB’s temporal evolution, and (h) shows how energy exchange among the spectral modes is altered in the presence of loss.

While most demonstrations of FPU recurrence within the AB framework have been conducted in weakly dispersive and nonlinear systems [102], there is limited research on how this phenomenon manifests in strongly dispersive and nonlinear systems, particularly on-chip optical devices. The USRN Bragg grating provides a unique platform to explore this behavior. In Figure 8, we excite an AB in the grating both without (left box) and with loss (right box) to observe FPU recurrence.

Without loss, as shown in Figure 8(a), the AB first emerges at *z* = 1 mm, with a second recurrence observed around *z* ≈ 2.5 mm. In Figure 8(b), the AB exhibits a narrow spectral bandwidth between recurrences, while the compression points display a comparatively broader bandwidth. This behavior arises from the grating’s strong dispersion, which reduces the MI bandwidth, limiting the number of excited sidebands [106]. The critical MI frequency parameter, $\omega_{c}^{2}=4\gamma P_{0}/|\beta_{2}|$ , highlights this effect, as large *β*_2_ values narrow the MI band. Including strong *β*_4_ further reduces the bandwidth, as predicted by Eq. (6). For this demonstration, we selected a pump wavelength of 1,543.57 nm within Region 1 of Figure 7(c), where the influence of higher-order dispersion on AB dynamics is minimal. This choice allows for a smooth observation of FPU recurrence.

In Figure 8(c), the AB’s amplitude trajectory is shown on a complex plane, with color representing the evolution distance *z*. The central trajectory corresponds to an undisturbed AB solution, while arrows indicate the flow of evolution. This is to differentiate how external perturbation impacts the FPU processes. The first half of the trajectory reflects the AB’s first emergence, and the second half represents its second recurrence. In an ideal NLSE system, subsequent AB recurrences would perfectly overlap this trajectory unless influenced by external dynamics. In contrast, the outer trajectory reflects the AB evolution from Figure 8(a), showing significant deviations due to strong nonlinearity and dispersion effects.

Figure 8(d) illustrates the energy exchange between the pump and discrete sidebands during the AB’s evolution. Taken from Figure 8(b), the plot highlights one side of the symmetric spectral profile. Downward black arrows indicate points where the pump’s energy is maximally transferred to the sidebands. The two nearest sidebands (in red and yellow) capture most of the power, while those below the black dashed line retain minimal energy, resulting in lower intensity.

In the right panel, we examine AB evolution with loss included. Figure 8(e) shows that loss delays the AB’s appearance, reduces its intensity, and introduces multiple low-intensity recurrences as the grating loses power. MI-driven growth is counteracted by the exponential loss term, *ω*_*c*_ = *ω*_*c*_ exp(−*α z*/2), creating a dynamic interplay. This competition produces a rapid oscillatory recurrence pattern, where AB power diminishes with each cycle. Figure 8(f) reflects these spectral dynamics, showing energy concentrated near the pump due to loss suppressing MI-induced gain, thereby reducing the effectiveness of four-wave mixing.

Building on the complex plane evolution, Figure 8(g) illustrates four orbits of the AB trajectory corresponding to four recurrence events observed in Figure 8(e). Temporal overlap among the recurrences is indicated by crossing points, marked by inward black arrows. This reveals a critical distinction in FPU recurrence behavior between conservative and non-conservative systems. In the absence of loss, the grating acts as a conservative system, where energy is preserved and transferred equally during each successive recurrence. However, when loss is introduced, the system transitions to a non-conservative state. Each successive recurrence inherits progressively less energy from the previous one, resulting in diminished intensity along the grating’s length.

Figure 8(h) highlights energy transfer among spectral components in the presence of loss. As significant power is lost during each recurrence, four-wave mixing weakens, leading to fewer and less intense sidebands over time. The decreasing sideband intensity, both above and below the dashed line, is indicated by a downward trend. Black arrows on the pump denote progressively weaker energy exchange, emphasizing the impact of loss on spectral dynamics.

**Figure 9:**
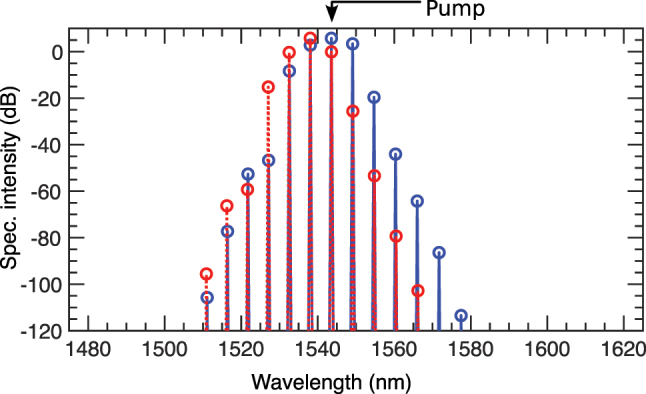
Spectral comparison at AB’s first maxima: The spectra without loss (blue) and with loss included (red) are compared.

To further analyze the effects of loss, Figure 9 compares the AB’s spectral profile at its first maximum compression point. The blue solid line represents the loss-free case, while the red-dashed line shows the profile with loss. With loss, the shorter wavelength side of the spectrum exhibits a slight gain, but there is a significant reduction in spectral power on the longer wavelength side. This asymmetric spectral profile indicates that TOD dominates for the pump at *λ*_0_ = 1,543.56986 nm, exciting fewer modes compared to the loss-free case.

### 4.6 Pattern formation and spectral enhancement in the grating

**Figure 10:**
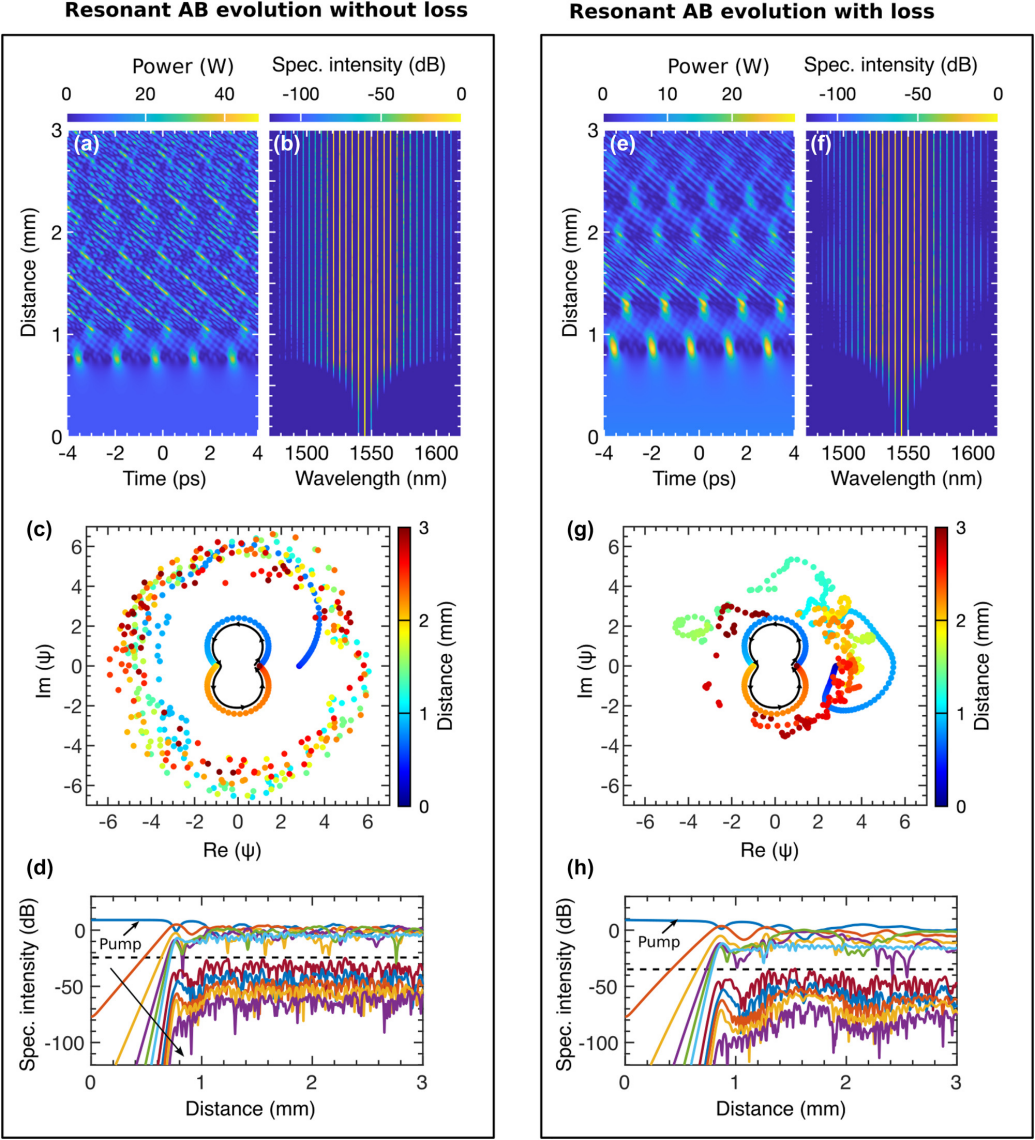
Pattern formation: [Left box – without loss] (a) temporal and (b) spectral evolution of an AB at pump wavelength *λ*_0_ = 1,545.35 nm, where the FPU-recurrence phenomenon disappears after the first growth-return cycle. (c) presents the amplitude evolution on a complex plane, with each dot representing a temporal peak along the propagation direction (*z*). The color gradient (blue to red) indicates progression along *z*, and increased scattering reflects greater amplitude variations. (d) shows energy exchange between the pump and sidebands, with the black dashed line separating highly amplified from moderately amplified sidebands. The colored spectral lines highlighted by the long downward arrow represent the first 10 sidebands respectively on the right side of the pump in (b). [Right box – with loss] (e) temporal and (f) spectral evolution of the same AB under loss. (g) amplitude evolution on a complex plane, where peaks are more resolved, indicating reduced amplitude variations. (h) illustrates energy exchange among the pump and sidebands, with loss-reducing amplification slightly.

In nonlinear systems, complex patterns arise from simple interactions, revealing structures and behaviors shaped by feedback loops and self-organization. Coherent and incoherent MI is crucial in forming patterns in instantaneous and non-instantaneous nonlinear systems, respectively [60], [107], [108]. In coherent MI, where the driving field is typically a monochromatic CW laser, the instability occurs due to the interaction between the medium’s nonlinearity and the small perturbations in the input field. This interaction leads to the exponential growth of sidebands around the input frequency, producing periodic intensity patterns within the beam. By leveraging this coherent MI, we demonstrate that the grating can generate complex patterns and induce parametric amplification in the spectral components of the driving field. In the left box of Figure 10, we present how the grating generates patterns and spectral amplification without loss, while the right box shows loss included.

In Figure 10(a), the temporal evolution of the pump field *λ*_0_ = 1,545.34998 nm appeared as an AB at *z* ≈ 0.75 mm. The presence of small positive *β*_4_ at this wavelength symmetrically creates additional MI sub-bands. One example of such MI bands is presented in Figure 4(c) with dotted blue lines (legend 5). The MI frequencies from these sub-bands strongly seed the MI process, leading to an enhanced phase matching among the pump and the new generating sidebands. This facilitates an efficient and sustained energy transfer from the pump to the new excited frequency modes. This, in turn, in the temporal domain, translates to the emission of strong background dispersive waves from the base of the AB. The generated dispersive waves are strong enough to sweep across the wave field and prevent the formation of new AB recurrences as the field advances in the grating length [82].

As a result of strong phase matching, in Figure 10(b), we observe a high-intensity spectral profile with many discrete frequency modes. Because there is no further development of AB in the *z* direction, we do not observe any FPU recurrence both in the temporal and spectral domains. From start to end, the wideband spectral intensity nearly remains the same. Although the speckle filaments in the temporal domain appear chaotic to some extent, they are strongly phase-matched. To elucidate how the patterns form, we provide a detailed explanation using the phase portrait of the temporal evolution in Figure 6 in the Supplementary material.

The reason behind the disappearance of the AB is pronounced on the AB’s complex plane trajectory of amplitude in Figure 10(c). In the outer orbit, the trajectory starts smoothly at *z* = 0 with dark blue. After a while, the scattered blue dots are indicative of the sudden deviation of the trajectory from the AB state to the multipeak background radiation waves. The highly scattered dots strongly indicate that the background waves have a diverse range of peaks. However, because they still remain within a well-defined region of an orbit means, until the evolution ends, the peaks repeat themselves a number of times, creating a pattern in the spatiotemporal domain. Note that, for a standard AB, the amplitude of the periodic pulse train smoothly changes in *z*. As a result, we observe a near continuous trajectory of its amplitude, such as in Figure 8(c) and (g).

Figure 10(d) shows that the pump strongly couples with the spectral modes. Sidebands closer to or farther from the pump are separated by a black dashed line. The intensity of the sidebands nearest the pump (above the dashed line) remains nearly equal to that of the pump itself. Notably, there is no periodic energy exchange in the sidebands, both above and below the dashed line, indicating that the AB no longer participates in the reccurence dynamics. This observation highlights that energy transfer from the pump to the sidebands is steady and robust rather than oscillatory, maintaining a nearly constant spectral intensity along the entire grating length.

High loss in the grating significantly impacts these dynamics, as shown in the right box. In Figure 10(e), the nonlinear interactions in the temporal evolution are now subdued, with the AB completely dissipating after two recurrences due to exponential power decay. The intense radiation waves seen in Figure 10(a) are now much weaker, and the background patterns are less pronounced. Interestingly, MI remains strong despite high loss, as evident in the spectral domain in Figure 10(f). Although power loss reduces spectral intensity somewhat, the overall spectral width remains unchanged. Notably, the intensity of the spectral sidebands near the pump is still comparable to the loss-free case shown in Figure 10(b).

Further insights come from the AB’s amplitude evolution on the complex plane in Figure 10(g). Here, the peaks are more resolved than those in Figure 10(c). Starting from dark blue, the trajectory in the outer orbit approaches the inward direction, with points clustering closer together, indicating fewer peak developments and a less pronounced temporal pattern.

In the energy exchange dynamics shown in Figure 10(h), the pump and its neighboring sidebands (above the black dashed line) retain high intensity, appearing less affected by loss. The sidebands farther from the pump (below the dashed line) maintain a relatively lower power level compared to the loss-free case in Figure 10(d). Remarkably, MI can strongly counteract the high loss at this pump wavelength, producing high-intensity, wideband spectral content at the output.

**Figure 11:**
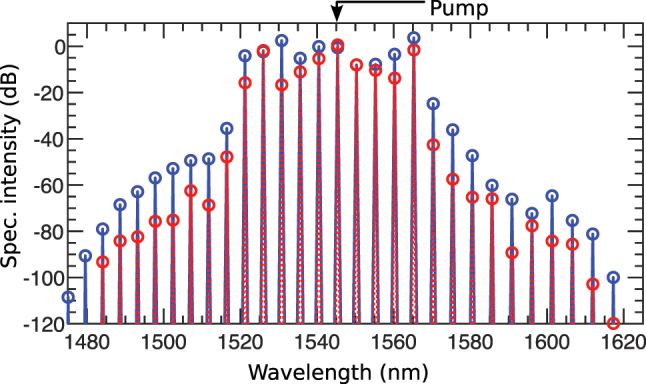
Grating’s output spectrum in pattern formation: The output spectrum is shown for the cases without loss (solid blue) and with loss (dashed red).

Finally, Figure 11 compares the output spectral profiles for the loss-free case (solid blue) and with loss (red dashed). Without loss, the shorter wavelength side of the spectrum gains more intensity than the longer wavelength side, reflecting asymmetry due to negative TOD. As previously shown in Figure 10(h), the sidebands near the pump exhibit similar intensity in both cases. This demonstrates the USRN Bragg grating’s potential for robust parametric amplification of spectral intensity, even under high-loss conditions.

## 5 Discussion

We theoretically investigated MI dynamics within the framework of the AB in a USRN Bragg grating characterized by complex dispersion curves and high nonlinear values. The wavelength-dependent nature of MI in this grating is particularly pronounced at the stop-band edge, where rapid variations in dispersion and nonlinearity introduce intricate dynamics. Each wavelength serves as a potential pump, and our numerical simulations revealed that only specific pump ranges generate new phase-matched frequencies, both continuous and discrete. This wavelength-dependent behavior emphasizes the critical role of dispersion and nonlinear profiles in determining the grating’s response to an input CW field.

Using linear stability analysis, we complemented numerical simulations to characterize how MI bandwidth and resonance wavelengths are influenced by variations in loss and power. We revealed that high power extends the range of accessible MI frequencies, while low power restricts it. Notably, we discovered that even with high loss, the grating still operates effectively at low power levels, as low as tens of milliwatts, enabling a new MI regime. However, we observed discrepancies between linear stability analysis and numerical simulations. Linear stability fails to account for TOD effects, which significantly impact MI dynamics, missing additional features in the output spectrum. Whereas, numerical analysis revealed those features, underscoring the necessity of combining both methods for a comprehensive analysis.

Using IST, we confirmed that noise-induced MI in a modulated CW field evolves into an AB-type solution within the grating. We identified wavelength ranges capable of exciting ABs, which generate discrete spectral profiles in the output. Interestingly, a significant portion of the nonlinear and dispersion profiles does not support AB development, as explained through insights from the standard AB solution and MI band characteristics. To showcase the grating’s versatility as a nonlinear platform, we demonstrate two novel nonlinear phenomena in the grating: FPU recurrence and complex pattern formation. We showed that the pattern formation in the grating is accompanied by wideband spectrum generation and parametric amplification.

We realize that the interaction of input light with the grating’s MI properties enables precise spectral control in the grating. These features make it an ideal platform for compact light sources and high-sensitivity on-chip sensors. Also, our analysis of the grating’s resonance structure reveals its potential as a tunable pulse generator. By tuning the CW pump laser from 1,539 to 1,546 nm, specific pump wavelengths can selectively generate new continuous and discrete frequencies. This tunability could be harnessed for applications in optical communications and photonic signal processing.

MI has historically been a cornerstone of nonlinear science, leading to significant discoveries such as solitons, optical rogue waves, FPU recurrence, pattern formation, supercontinuum generation, and parametric amplification. Despite these advancements, the exploration of MI in on-chip Bragg gratings remains relatively underdeveloped. Optical rogue waves – intense, spontaneous bursts of light driven by MI [72] – are of particular interest due to their analogy with oceanic rogue waves [109] and their role in various extreme instability-driven phenomena [24]. This work investigates the feasibility of studying MI in USRN Bragg gratings, focusing on the power and length requirements for experimental realization. Our findings provide a foundation for future studies of MI-induced optical rogue waves in compact photonic platforms, bridging the gap between fundamental nonlinear wave dynamics and practical on-chip applications.

More importantly, the rogue wave phenomena have been recognized as a crucial mechanism for noise suppression in supercontinuum generation, significantly enhancing spectral coherence and stability [110], [111]. From this perspective, controlling noise in an integrated platform such as USRN Bragg gratings is particularly promising. The ability to engineer MI in these gratings provides a controlled environment for studying rogue wave formation and its impact on supercontinuum generation, offering new strategies for achieving ultra-low-noise optical sources. This is especially relevant for the development of integrated photonic quantum technologies, where precise control over spectral purity and noise reduction is critical [112], [113]. Our study of MI, combined with its demonstrated experimental feasibility in USRN Bragg gratings, establishes a viable pathway toward realizing noise-stable, high-coherence quantum photonic devices on a compact, chip-scale platform.

Furthermore, control over MI and rogue waves plays a pivotal role in enhancing supercontinuum generation, a process fundamental to numerous optical applications, including pulse amplification, compression, sensing, switching, and coherent light control. While supercontinuum generation has been extensively studied in optical fibers [114], it remains relatively less explored in chip-scale Bragg gratings. The USRN Bragg grating has already been experimentally employed as a pulse compression device in a USRN waveguide [54], [68], demonstrating its capability to generate a supercontinuum spectrum. To achieve optimal performance and produce high-quality spectra from supercontinuum generation in these devices, a deeper understanding of the underlying pulse dynamics is essential. Our investigation into MI and AB dynamics in the USRN Bragg grating offers valuable insights into this context, providing a foundation for advancing the study of on-chip supercontinuum generation. By elucidating the interplay between dispersion, nonlinearity, and MI in the grating, this study highlights the potential for precise spectral control, which is essential for next-generation integrated photonic devices.

To underscore the importance of this platform, we present Table 1, which compares MI in this system (highlighted column) with other optical platforms. Note that because MI depends on multiple system parameters, including power, nonlinearity, dispersion, and loss, the input power and pulse duration for MI onset also vary across different platforms. As a result, instead of providing exact values, the table presents approximate ranges for these parameters. Giving precise values would be challenging due to the complex interplay of factors influencing MI dynamics.

**Table 1:** Comparison of MI in various optical systems.

Parameter	USRN Bragg grating	Silicon (waveguides)	Silicon nitride (waveguides)	Chalcogenide (waveguides)	Silica fiber	Fiber Bragg gratings	Gas-filled Hollow-core fiber	Highly nonlinear fiber
Length scale	1–3 mm (compact)	1–10 mm	1–10 mm	10 mm–20 cm	100’s of m–km	10–15 cm	few cm–m	m–km
Power levels	10s of mW–10 W	10s of mW– 10 W	10s of mW–10 W	mW–W	W–kW	W	mW–W	mW–W
Minimum pulse widths	100’s fs–10’s ps	100’s fs–ps	100’s fs–ps	100’s fs–ps	ps–ns	ps–ns	fs–ps	fs–ps
Dispersion engineering	Tailorable	Tailorable	Tailorable	Tailorable	Limited	Tailorable	Tailorable	Tailorable
Nonlinear coefficient	$∼10^{6}$ W^−1^km^−1^	$∼10^{5}$ –10^4^ W^−1^km^−1^	$∼10^{3}$ W^−1^km^−1^	$∼10^{4}$ W^−1^km^−1^	∼1 –10 W^−1^km^−1^	∼1 –10 W^−1^km^−1^	∼1 –10 W^−1^km^−1^	∼10 –100 W^−1^km^−1^
Platform advantages	Compact,	Compact,	Compact,	High nonlinearity	Long interaction	Filtering	Gas tunability	Very high
	CMOS-compatible,	CMOS-compatible,	CMOS-compatible					nonlinearity
	& very high	and high						amongst fibers
	nonlinearity	nonlinearity						
References	[53], [55], [115], [116]	[117], [118], [119]	[50], [120]	[121]–[123]	[3], [4], [79]	[47], [48], [124]	[125], [126]	[114], [127], [128]

Separated by the thick black line in the middle, the table highlights key differences in MI development across fiber-based and on-chip waveguide systems, focusing on several critical MI parameters such as length scale, pulse duration, and power requirements. Fiber-based systems (e.g., silica, highly nonlinear fiber) require long interaction lengths ( ∼10 ’s of cm–100’s of m or even Km), higher power (W–kW), and longer pulses (100’s of fs–10’s of ps or even ns) due to their lower nonlinearity and longer length requiremnt to accmulate effective dispersion strength. However, on-chip waveguides (e.g., Si, SiN, chalcogenide) enable MI over significantly shorter lengths (a 10 mm–5 cm) and at lower power levels (10s of mW–a few W) due to stronger confinement and higher nonlinear coefficients, but they often face fabrication and dispersion constraints.

Among these platforms, USRN Bragg gratings offer superior performance by combining higher nonlinearity, comparatively lower loss, and enhanced dispersion engineering [53], [55]. Unlike standard SiN and Si waveguides, USRN enables MI to develop over much shorter lengths while maintaining comparable power and pulse widths requirements. Bragg gratings in USRN further improve dispersion control and introduce slow-light effects, significantly lowering power and length requirements while enabling broader MI gain bandwidths within just 1–2 mm grating lengths. This makes USRN more efficient than Si, SiN and chalcogenide waveguides and more compact than fiber systems, supporting higher-order MI dynamics, optical rogue waves, and ultra-low-noise supercontinuum generation.

Despite its promise, like other chip-based platforms, the grating exhibits limitations due to its loss properties. In optical fibers, low loss ensures robust MI dynamics, but the USRN Bragg grating’s high loss poses challenges for sustained MI development along its full length. While the grating’s strong nonlinearity and dispersion initiate MI early, loss suppresses further MI growth, resulting in less intense spectral content at the output. However, at specific resonance frequencies, MI overcomes loss-induced power decay, enabling the generation of high-intensity new frequencies far from the pump. This demonstrates the grating’s resilience and versatility, even under high-loss conditions. Although our theoretical studies employed comparatively high power (*P*_0_ = 8 W) for clarity, many observed dynamics persist at lower power levels, albeit with reduced spectral bandwidth or intensity.

Another important consideration is whether the dispersion varies along the propagation direction (z) and whether this variation influences the nonlinear dynamics in the grating. In our study, we assume an effective dispersion profile that is uniform along the grating length. This assumption is justified because the apodized section comprises only a small portion of the overall device (typically, the apodized input section constitutes 3 % of the total grating length), and thus, the propagation is primarily governed by the uniform region. Moreover, the gradual reduction in bandgap strength within the apodized region further minimizes its impact on wave propagation. Consequently, the primary nonlinear dynamics remain qualitatively unaffected. This assumption is consistent with previous experimental studies [129], where uniform grating models accurately predicted the nonlinear pulse behavior in apodized structures. Nonetheless, future investigations incorporating a fully z-dependent dispersion model could provide a more detailed understanding of these effects.

Beyond integrated photonics, MI, FPU recurrence, and pattern formation provide insights into emergent phenomena in natural systems, such as fluid patterns, brain waves, and biological structures. Studying these phenomena in a Bragg grating contributes to the broader understanding of their manifestations across diverse systems. While our work highlights key nonlinear interactions in the grating, several areas remain unexplored. For instance, further investigation is needed into FPU recurrence under loss and other perturbations, where oscillatory trends were observed but not fully explained. Additionally, studying AB behavior under different MI regimes and exploring higher-order MI and AB phenomena in this grating present exciting future research opportunities.

This study represents a significant step toward understanding complex nonlinear dynamics in integrated photonic platforms. By bridging theoretical insights with practical applications, we aim to inspire further research into the capabilities and limitations of chip-scale Bragg gratings, ultimately advancing the field of integrated photonics.

## 6 Methods

*Simulation:* The values of *γ*, *β*_2_, *β*_3_, and *β*_4_ in Figure 2(a) and (b) are experimentally measured over a wavelength range from 1,539 nm to 1,546 nm, sampled at 4,991 discrete points. Simulating the full dataset with wavelength-dependent dispersion and nonlinear parameters is computationally demanding. Therefore, we select every 5th data point, resulting in a reduced set of 899 wavelengths. This downsampling preserves the overall shape and features of the *γ*, *β*_2_, *β*_3_, and *β*_4_ curves without significant loss of information. We then simulate the MI dynamics for these 899 discrete wavelengths using their corresponding measured dispersion and nonlinear parameters. The resulting phase-matching behavior is presented in Figure 2(c). The GNLSE is solved using the standard split-step Fourier method.

*Linear stability analysis:* To conduct a linear stability analysis, we need to solve the normalized generalized nonlinear Schrödinger equation (GNLSE) which can be given by [3]:

(7) $$i\frac{\partial \psi }{\partial z}-\frac{\beta_{2}}{2}\frac{\partial^{2}\psi }{\partial t^{2}}+|\psi {|}^{2}\psi =\delta_{3}\frac{\partial^{3}\psi }{\partial t^{3}}+\delta_{4}\frac{\partial^{4}\psi }{\partial t^{4}}$$

where $\delta_{3}=\frac{\beta_{3}}{6|\beta_{2}|T_{0}}$ , $\delta_{4}=\frac{\beta_{4}}{24|\beta_{2}|T_{0}^{2}}$ . *T*_0_ is the input pulse width.

The GNLSE admits a steady-state solution in the form of:

(8) $$\psi =\sqrt{P_{0}}\exp\left(iP_{0}\gamma z\right)$$

To assess the stability of this solution, the amplitude is perturbed at frequency *ω*, modifying the steady-state solution to:

(9) $$\psi =\left[\sqrt{P_{0}}+a\left(z,t\right)\right]\exp\left(iP_{0}\gamma z\right)$$

where *a*(*z*, *t*) = *a*_1_ cos(*kz* − *ωt*) + *ia*_2_ sin(*kz* − *ωt*). Solving Eq. (7) under these conditions yields the dispersion relation given by:

(10) $$k=\frac{1}{2}\left(\sqrt{B_{2}+B_{4}}\right)$$

Here, *B*_2_ and *B*_4_ are defined as follows:

(11) $$B_{2}=\omega^{2}\beta_{2}^{2}\left(\omega^{2}+\omega_{c}^{2}\right),$$

(12) $$\;B_{4}=\left(\beta_{4}\omega^{4}/144\right)\left[12\beta_{2}\left(2\omega^{2}+\omega_{c}^{2}\right)+\omega^{4}\beta_{4}\right]$$

with $\omega_{c}^{2}=\left(4\;P_{0}\;\gamma \right)/\beta_{2}$ . The dispersion relation in Eq. (14) clearly illustrates the relative influences of the group velocity dispersion parameter *β*_2_ and the fourth-order dispersion *β*_4_. The stability of the initial plane wave is determined by the reality of *k*, where instability occurs only when *k* is imaginary. It is essential to highlight that, under specific conditions (*γ* = *P*_0_ = 1, *β*_2_ = −1, resulting in $\omega_{c}^{2}=-\;4$ ), Eq. (14) represents the dispersion relation for the normalized form of Eq. (7) [3], [130]. This specific form is utilized in our analysis. Furthermore, when *β*_4_ = 0, Eq. (14) yields $k=\omega /2\;\sqrt{\left(\omega^{2}-4\right)}$ , providing the gain expression directly in AB solutions as presented in [98], [105]. For *β*_2_ < 0 and *β*_4_ = 0, *k* becomes imaginary when $\left(\beta_{2}+\beta_{4}\;\omega^{2}/12\right)<0$ and

$$\left(\beta_{2}+\beta_{4}\;\omega^{2}/12\right)\left(\omega^{2}/\beta_{2}\right)<\omega_{c}^{2}$$

with these conditions, the gain can be given as [3], [81], [131]:

(13) g(ω)=Im(k)

It is important to note that *β*_3_ is omitted in the investigation of MI through linear stability analysis. For phase matching in a four-wave mixing process, the *β*_3_ term cancels out, as shown in [3] and [131] (see Eq. 10.1.7), leading to *β*_3_ = 0. Although the theory of an AB establishes that odd-order dispersion introduces velocity in the pulse profile while even-order dispersion influences MI and the phase of the pulse profile [132], it is crucial to clarify that the literature sometimes mistakenly suggests that third-order dispersion (TOD) does not contribute to MI dynamics at all. Contrary to this misconception, several works, such as [77], [133], [134], have presented numerical studies on TOD’s contribution to MI. For numerical simulations, we employed the standard split-step Fourier method in conjunction with the fourth-order Runge-Kutta method to solve Eq. (7) [135].

*IST analysis:* To prove that the extracted wave envelope shown in Figure 6(e) is indeed an AB-type solution, we performed the IST spectral analysis. Although our master equation is GNLSE, however, the IST spectral analysis is done within the framework of integrable NLSE which can be presented as the compatibility condition of the two linear equations called the Lax pair [76]:

(14) $$\Phi_{t}=\left(\begin{array}{cc} i\lambda & \psi \\ -\psi^{*} & -i\lambda \end{array}\right)\Phi$$

(15) $$\begin{array}{ll} \Phi_{z} & =\left(\begin{array}{cc} -i\lambda^{2}+\frac{i}{2}|\psi {|}^{2} & \lambda \psi +\frac{i}{2}\psi_{t}\\ -i\lambda \psi^{*}+\frac{i}{2}\psi_{t}^{*} & i\lambda^{2}-\frac{i}{2}|\psi {|}^{2} \end{array}\right)\Phi \end{array}$$

For the IST analysis, we only required the spatial part of the Lax pair where *λ* represents the eigenvalues *Y* is an eigenvector and the subscript ‘∗’ indicates the complex conjugate. Before obtaining the eigenvalues, Eq. (14) is transformed into an eigenvalue problem for eigenvalue *λ* such that:

(16) $$\hat{L}\left\{\Phi \right\}=\lambda \Phi ,\;\;\hat{L}=i\left(\begin{array}{cc} 1 & \;0\\ 0 & -1 \end{array}\right)\frac{\partial }{\partial_{t}}-i\left(\begin{array}{cc} 0 & \psi \\ \psi^{*} & 0 \end{array}\right)$$

where $\hat{L}$ is the Zakharov–Shabat operator and $\Phi ={\left\{\phi_{1}\left(t\right),\phi_{2}\left(t\right)\right\}}^{T}$ is the eigenvector. The Zakharov–Shabat operator with the eigenvector operates on the potential *ψ* in the eigenvalue problem. The solution to the problem provides the corresponding eigenvalues *λ*.

For a numerical calculation of these eigenvalues, an arbitrarily selected localized structure from the chaotic wave field as potential is defined within a numerical box with a size of *L*. This is then discretized with a large number of points *n* ≈ 2^8^ − 2^16^, the higher the points, the better the accuracy and resolution of the selected wave profile. The eigenvector and the potential are expressed in Fourier series using 2*n* + 1 number of modes. This Fourier expansion of the potential is utilized in Eq. (14) and solved for the eigenvalue *λ* using the Fourier collocation method [136]. These are a set of discrete IST spectrums of the selected localized part from the envelope *ψ* in Figure 6(e).

When the modulated CW evolves in the grating, it creates a highly chaotic wave field where various localized structures emerge. In this transient wavefield, new structures are created, and they seamlessly transform from one to another [137], [138], [139]. They are classified as radiation waves, plane waves, solitons, or AB-type solutions. For an accurate description of the evolution, it is important to know the type of structures in the wave field, which is a challenging task. Recently, a local periodization procedure [94] has been proposed to address this issue and we used this technique in our IST analysis.

## Supplementary Material

Supplementary Material Details
