# Supplementary material for: Theoretical studies of modulation instability, Fermi–Pasta–Ulam recurrence and pattern formation in an ultra-silicon-rich-nitride Bragg grating
Source: Nanophotonics. 2025 May 22;14(13):2267–94. doi: 10.1515/nanoph-2025-0073 (PMC12199563; doi:10.1515/nanoph-2025-0073)
Supplement: Supplementary file 1 — Supplementary Material Details [file j_nanoph-2025-0073_suppl_001.pdf]

Modulation instability, Fermi-Pasta-Ulam recurrence and pattern formation in an ultra-silicon rich nitride Bragg gratings

Amdad Chowdury[1,*] Benjamin J. Eggleton[2], and Dawn Tan[1,3†]

[1] *Photonics Devices and Systems Group, Singapore University of Technology and Design 8 Somapah Rd., Singapore 487372, Singapore*

[2] *Institute of Photonics and Optical Science, School of Physics, The University of Sydney, Sydney, New South Wales 2006, Australia and*

[3] *Institute of Microelectronics, Agency for Science, Technology, and Research (A*STAR), 2 Fusionopolis Way, Singapore 138634*

A. Temporal and spectral evolution at the edge of the region 1 and 3

We observe the spectral evolution at various resonance wavelengths in Fig. 2(c) of the main article, focusing on the spectral properties of the grating at specific regions and resonance wavelengths. While the main article emphasizes these spectral characteristics, the temporal evolution of the CW pump offers critical insights into the development of chaotic wave fields within the grating. The USRN Bragg grating, with its extreme dispersion near the stopband and large nonlinear coefficient, is particularly well-suited for studying these dynamics. Understanding how strong dispersion and nonlinear values influence the time development of ABs is crucial for optimizing the device's performance and for broader insights into MI dynamics in other Kerr nonlinear materials with significant dispersion. To facilitate this understanding, all temporal plots presented here show the amplitude evolution to highlight the AB's structural features.

To examine temporal behavior, we analyze examples from two distinct regions of the resonance structure: the bottom and the top, marked by black arrows b and f in Fig. 1(a). Beyond these resonance wavelengths and special regions 1, 2, and 3, the noise-induced evolution of ABs closely resembles the dynamics shown in Fig. 6(d) of the main article. For the first example, at a pump wavelength of 1540.68 nm, the temporal amplitude evolution is shown in Fig. 1(b). In the top panel of Fig. 1(c), we compare the amplitude of two transverse profiles: one (blue) taken at the AB's maximum compression point (white dashed line) and the other (brick-red) at the grating's output. The comb-like temporal profile (blue) indicates that the AB is highly compressed, whereas the brick-red profile highlights the grating's low amplitude output due to power dissipation from loss.

At this pump wavelength, multiple MI bands are present, as shown in Fig. 4(a) of the main article. These multiple MI bands seed the AB, generating a sea of scattered radiation waves. Because these waves span a broad frequency range, the frequency domain in Fig. 1(d) reveals a wide spectral bandwidth. Notably, two specific frequencies, marked by white arrows on either side of the pump, are amplified more than the others, clearly indicating the influence of multiple MI bands. The top panel in Fig. 1(e) compares the transverse spectral profile with the grating's transmission band, showing that the spectral width exceeds the transmission band. Even in the presence of loss, the spectral intensity remains sufficiently high for practical applications.

In contrast, pump wavelengths closer to the stopband produce ABs with narrower output frequencies, even with multiple MI bands, as demonstrated at a pump wavelength of 1545.35 nm. In Fig. 1(f), the amplitude evolution differs qualitatively from Fig. 1(b). An AB develops at $z \approx 1.19$ mm, marked by the dashed white line. However, due to the strong dispersion and noise-modulated background, the AB is incoherent. As the evolution progresses, the AB disintegrates into narrow-band radiation waves. This behavior arises because the multiple MI frequency bands at this pump wavelength (see Fig. 4(a) in the main article) seed the AB development. Unlike the highly scattered waves in Fig. 1(b), the frequency content in Fig. 1(f) is less scattered due to fewer contributing frequency modes. The top panel in Fig. 1(g) compares the transverse profile (blue) at the AB's compression point with the output profile (brick-red). The wider temporal width of each periodic pulse across the AB reflects the narrower spectral bandwidth. The brick-red profile also highlights the reduced intensity at the grating's output.

In the spectral domain, Fig. 1(h) shows the emergence of a narrow bandwidth AB. Comparing the spectral evolution between Fig. 1(d) and Fig. 1(h) reveals a clear difference in frequency bandwidth when the pump is positioned away from or closer to the grating's stopband. The transmission band demonstrates that, in both cases, specific frequency components are allowed to propagate to the grating's output. These results underscore the importance of dispersion and nonlinear parameter tuning in tailoring the grating's temporal and spectral responses.

* amdadul.chowdury@sutd.edu.sg

† dawn_tan@sutd.edu.sg

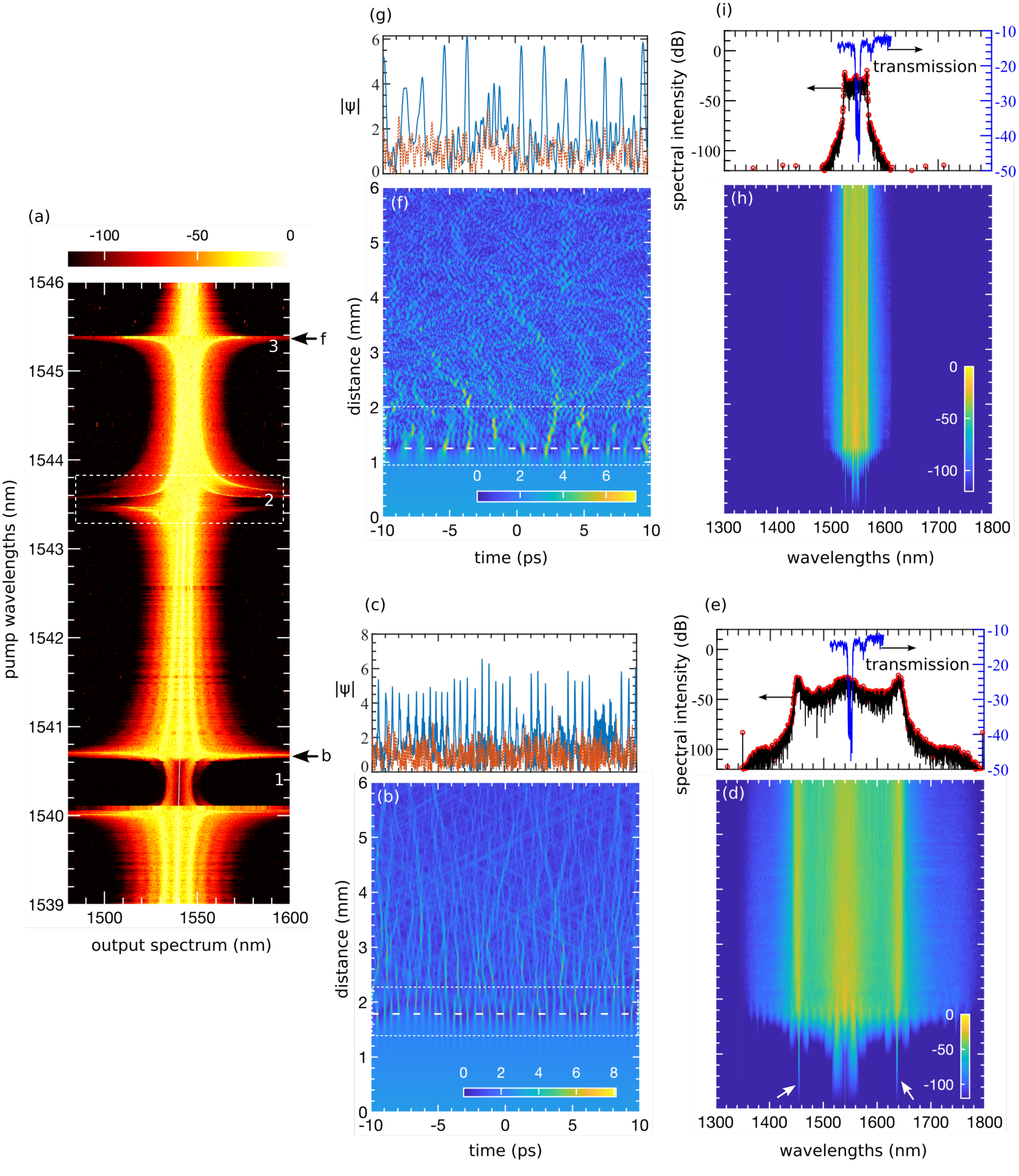

FIG. 1: **Evolution of a Noise-Induced AB Away and Closer to the Stopband.** **a** The resonance structure of the grating. **b** Temporal evolution away from the grating's stopband at 1540.68 nm, marked with a black arrow in **a**. **c** Two transverse profiles: one (blue) taken across the white dashed line within the white box in **b**, and another (brick-red) at the end of the grating. **d** Spectral evolution of **b**. **e** Transverse spectral profile (black) at the grating output, with the transmission band shown in blue. **f** Temporal evolution closer to the stopband at 1545.35 nm, indicated by a black arrow at the top of **a**. **g** Two transverse profiles from **f**: one (blue) across the white dashed line within the white box in **f** and another (brick-red) at the end of the grating output. **h** Spectral evolution of **f**. **i** Transverse spectral profile (black) at the output of the grating, with the transmission band shown in blue.

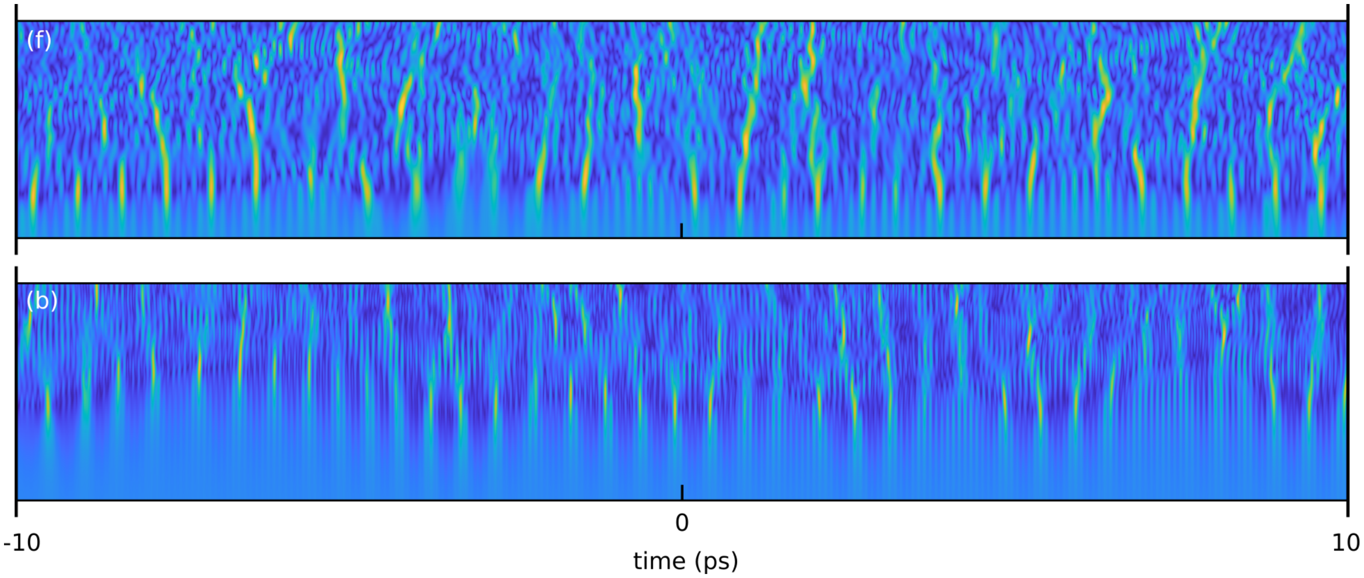

FIG. 2: **Enlarged view of the region highlighted by the white box in panels b and f of Fig. 1.**

To have a better visual, we enlarged the temporal evolution parts from Fig. 1(b) and Fig. 1(f) in Fig. 2. This zoomed-in portion clearly shows how strong dispersion and large nonlinearity distort the background and intensely impact the AB's appearance. Particularly, the emergence of parallel ripple within the AB's local profile is significant. Both of the pump wavelengths have multiple MI bands, and MI frequencies from them seed the development of the ABs. However, the seeding is so strong that they develop multi-wavelength strong radiation waves in the background, which works against the development of ABs themselves. This is why, in the forward evolution, we do not see any recurrence of them. In a similar scenario in optical fiber, where dispersion and nonlinear values are significantly lower than this grating, we do not observe this highly deformed background nonlinear interactions. It appears that these background features in Fig. 2 are unique to the grating's nonlinear and dispersion properties. We will discuss this part in more detail in the Fig. 6.

B. Dependence of MI bandwidth on power variations

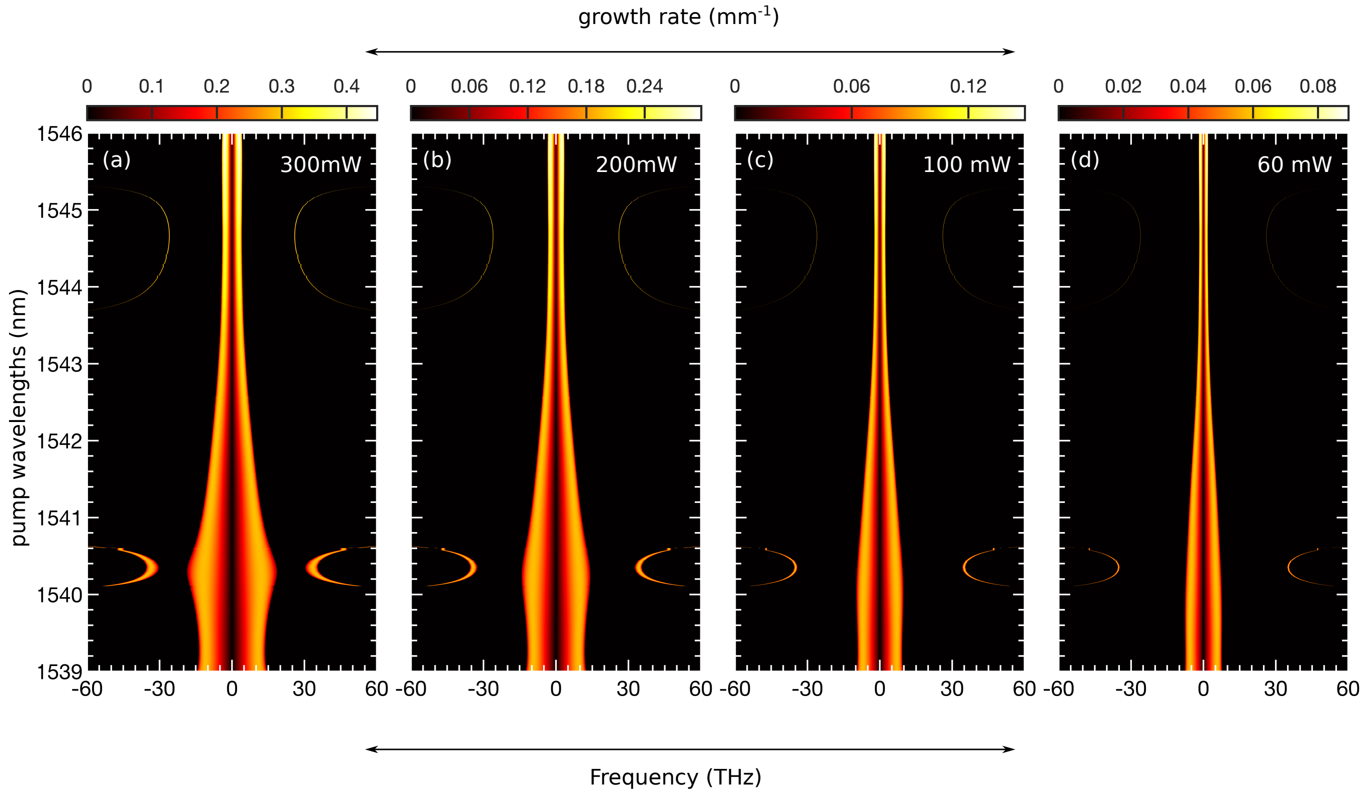

FIG. 3: **Variation in resonant MI frequency bandwidth and growth rate with power:** Panels **a**, **b**, **c**, and **d** correspond to powers of 300 mW, 200 mW, 100 mW, and 60 mW, respectively, with grating loss included.

Using linear stability analysis in the main article, we demonstrated how loss and power impact MI bandwidth. However, considering the practical implications, we present a few more examples in Fig. 3 showing how power level impacts MI bandwidths and ranges. In integrated photonics experiments involving optical components such as Bragg grating and similar devices, the laser power variations range from a few watts to in the order of tens of mW. In Fig. 3(a)-Fig. 3(d), we varied the power level from 300 mW to 60 mW. We observe that with decreasing power levels, the MI sub-bands near (top part of the pump wavelengths) the grating's stopband are disappearing rapidly compared to those that are away (bottom part of the pump wavelengths) from the stopband. This highlights that for high-power laser, one has access to resonant wavelength with multiple MI bands both away or closer to the stopband. However, if the laser power is low, the MI subband remains weaker closer to the stopband and still has access to those MI sub-bands that are away (bottom) from the stopband.

C. IST analysis for the full envelop across an AB

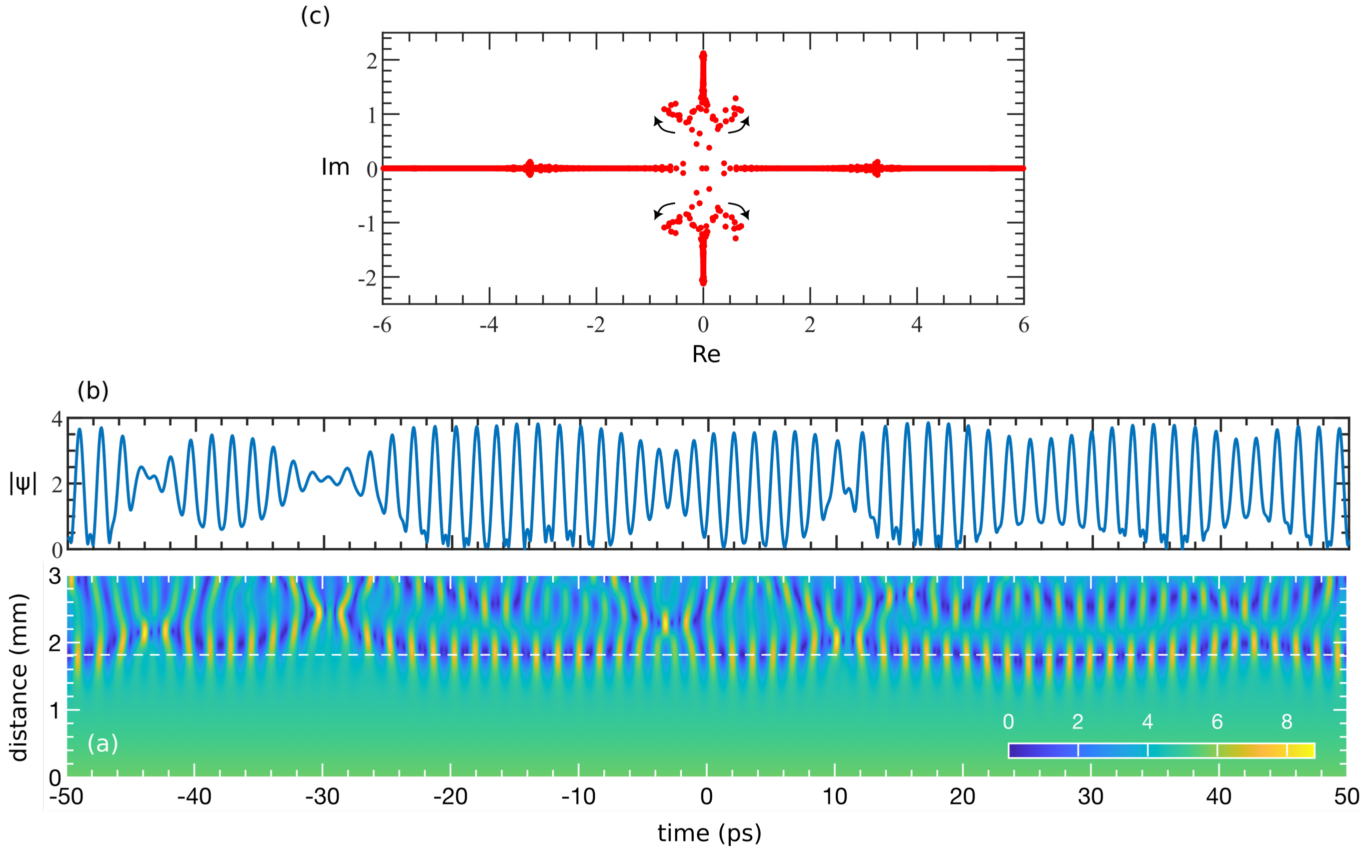

FIG. 4: **IST analysis confirming the emergence of ABs in the grating (bottom to top): a** Temporal amplitude evolution. **b** Transverse profile along the white dashed line in **a**. **c** IST spectra of the entire envelope shown in **b**.

In the main article, we selectively performed an Inverse Scattering Transform (IST) analysis on individually chosen pulse profiles extracted from the full envelope. Each selected profile was identified as a genus -2 solution, classifying them as Akhmediev Breather (AB) solutions. Notably, when the IST analysis is applied to the entire envelope, the signature of the AB solution remains evident. More importantly, the eigenvalues obtained from the full-envelope analysis reveal the interaction between the ABs and the background optical field. Figure 4(a) reproduces Figure 6(d) from the main article for reference. The amplitude of the full envelope along the white dashed line is extracted and presented in Figure 4(b), with the corresponding eigenvalues shown in Figure 4(c). In this analysis, the real axis represents the background radiation wave, while the eigenvalues aligned along the imaginary axis correspond to the AB solutions [1].

In an ideal scenario, devoid of higher-order dispersion and nonlinear effects, the eigenvalues of a standard AB solution extend uniformly along the imaginary axis up to 1 (see Fig. 6(c) in the main article). However, in this study, we utilize a non-ideal Nonlinear Schrödinger Equation (NLSE) model incorporating higher-order dispersion and strong nonlinear effects. These factors significantly influence the AB dynamics, leading to eigenvalues deviating from the imaginary axis and clustering near 1 in a scattered manner. This deviation indicates strong interactions between the ABs and the background wave, inducing asymmetry in the individual pulse profiles [2]. The black arrows in Figure 4(c) highlight that the eigenvalues, although scattered, align along spectral bands on the imaginary axis, as observed in the main article (see the bottom panel of Fig. 6). The eigenvalue distribution in both left and right directions confirms the persistence of asymmetry, covering all spectral band arrangements reported in the main article. It is important to note that not all eigenvalues exclusively correspond to AB solutions; some may indicate the emergence of Kuznetsov-Ma solitons [3]. Eigenvalues related to these solutions can extend beyond 1 along the imaginary axis [1].

D. Individual response

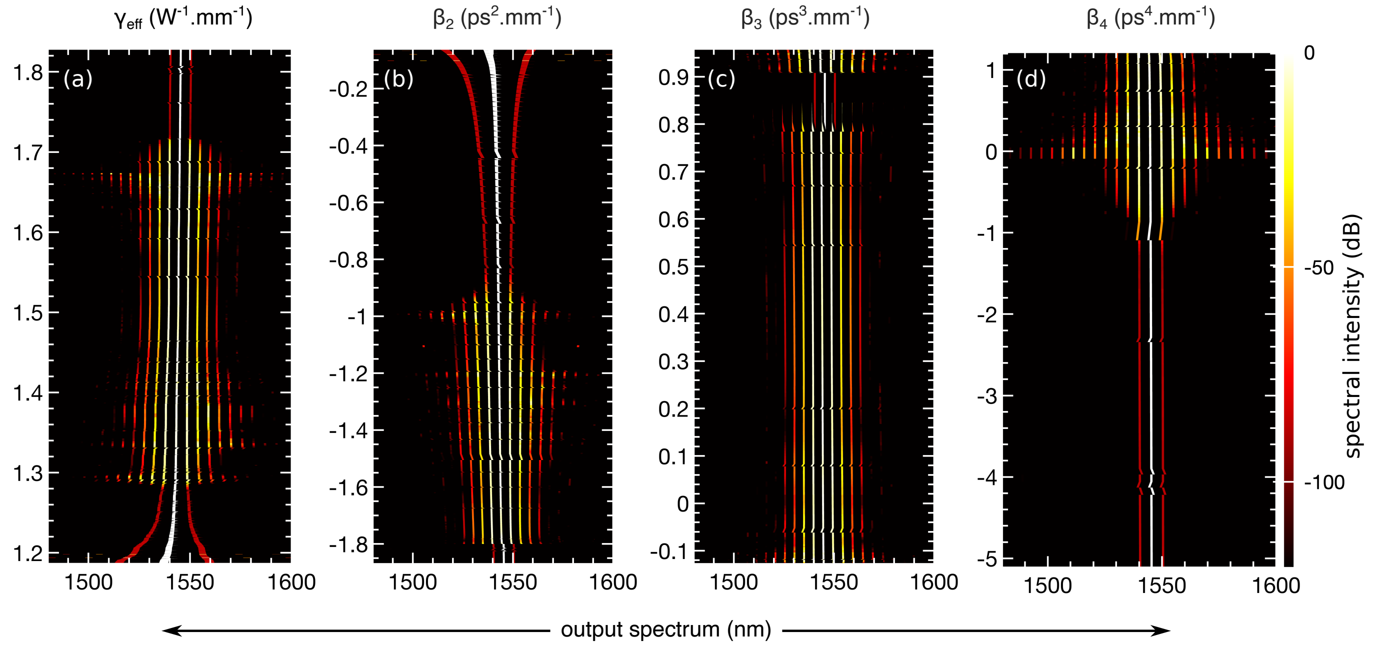

FIG. 5: **Individual contributions of nonlinear and dispersion parameters to the total resonance structure of the grating (The X-axis represents the output spectra, and the Y-axis represents the nonlinear and dispersion parameters.): a** Effective nonlinearity. **b**, **c**, and **d** show the contributions from β_2, β_3, and β_4, respectively.

Figure 5 illustrates the individual contributions of the nonlinear and dispersion parameters when a continuous wave (CW) is modulated at a modulation instability (MI) frequency. This analysis parallels the results presented in Fig. 3 of the main article. The nonlinear effect and third-order dispersion (β_3) remain active across most of their respective spectral ranges. Notably, the nonlinear parameter becomes inactive in two wavelength intervals: approximately 1539–1540 nm and 1545.5–1546 nm. In contrast, the second-order dispersion parameter (β_2) remains active only within the narrower range of approximately 1543.4–1545.6 nm, which closely aligns with the active range of the fourth-order dispersion parameter (β_4). This overlap between the active ranges of β_2 and β_4 suggests their combined contribution to the generation of resonance frequencies in the grating. A comparison with the resonance wavelengths depicted in Fig. 7 of the main article reveals a similar active spectral region. This correspondence underscores the critical role of β_2 and β_4 in determining the resonance wavelengths.

E. Time and phase evolution during pattern formation

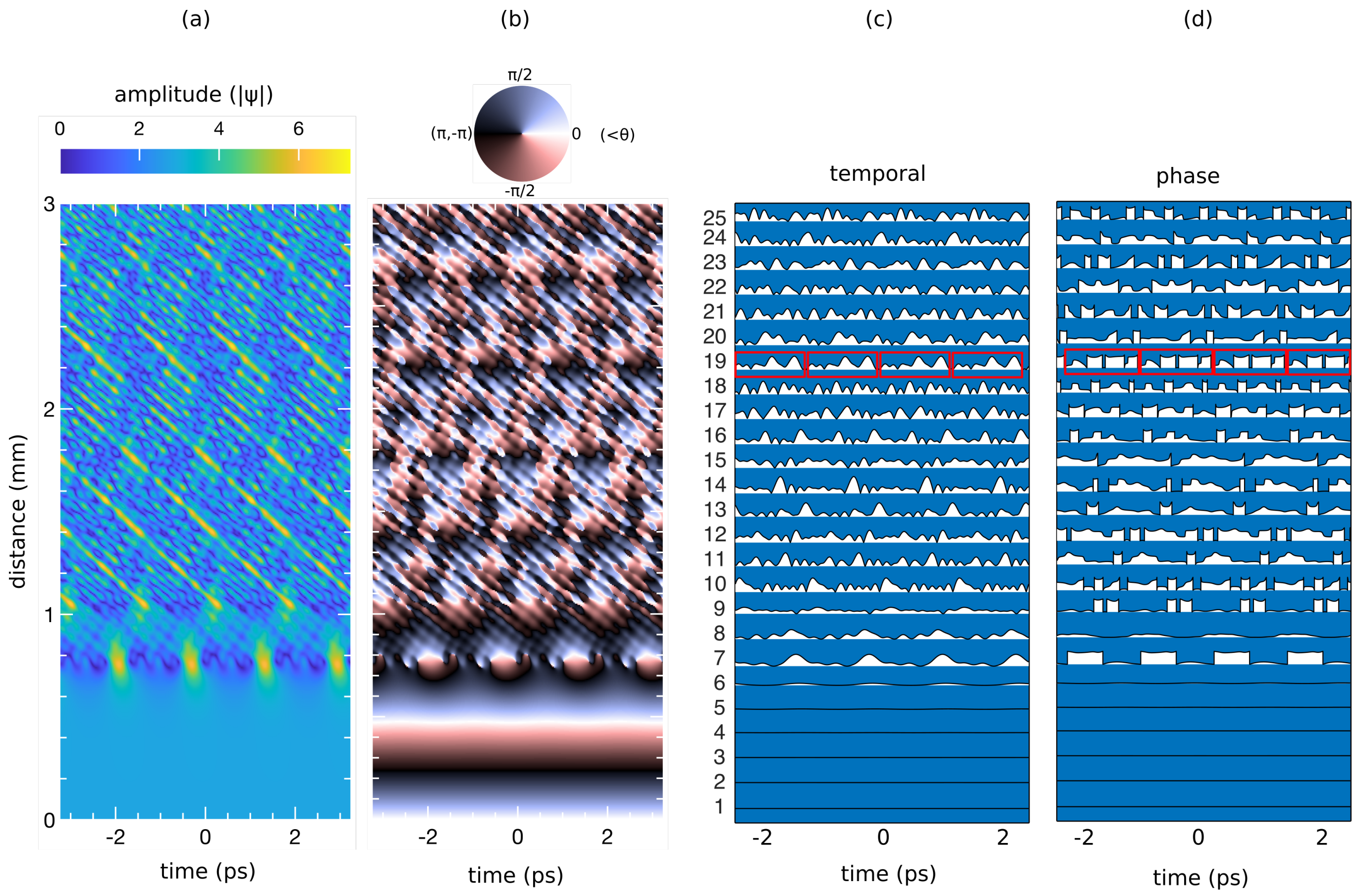

FIG. 6: **Demonstration of pattern formation: a** Temporal evolution at 1545.35 nm, identical to Fig. 10(a) in the main article. **b** Phase evolution. **c** and **d** show 25 intermittent transverse frames extracted from **a** and **b**, respectively. The phase of each temporal frame in **c** corresponds to the same frame in **d**.

From the temporal and spectral evolution, understanding the internal mechanism of pattern formation remains challenging. Figure 6 illustrates how the early-stage development of the Akhmediev Breather (AB) contributes to the formation of patterns along the extended evolution direction. In Fig. 6(a), we present the temporal evolution of the amplitude, showing the first appearance of the AB at z = 0.75 mm. The phase evolution, displayed in Fig. 6(b), reveals the characteristic periodic phase shifts of the AB, alternating between 0 and π. However, as evolution progresses, strong background radiation sweeps through the field, disrupting the recurrence of the AB. Instead of repeated ABs, we observe complex and regular patterns forming in both the temporal and phase domains, which are difficult to interpret directly from the evolution data.

To simplify the interpretation, we decompose the evolution into 25 intermittent transverse profiles and plot them in Fig. 6(c) and 6(d). The temporal profiles in Fig. 6(c) are directly related to the phase profiles in Fig. 6(d). For example, the AB appearing at $z = 0.75$ corresponds to frame 7, where the temporal and phase profiles show the periodic structure of alternating π and 0 phase shifts. Although the AB's structure is slightly distorted, its periodic phase structure remains intact. Even though the AB loses its recurrence property and disappears along the evolution (z) direction, its periodicity in the transverse dimension is preserved. This transverse periodicity is crucial for generating consistent patterns in the overall spatiotemporal domain.

The periodicity of the AB pulses in the transverse dimension is $n(2\pi/\omega_{\text{mod}})$, where n represents the number of pulses within the AB and ω_{mod} is modulation frequency. To highlight this preserved periodicity, we examine frame 19 in Fig. 6(c), where each pulse is enclosed within a red box. The corresponding periodic phase changes are shown in the same frame in Fig. 6(d), also marked within red boxes. The distortion observed in these pulses arises from strong seeding by multiple frequencies within the modulation instability (MI) subbands near the pump wavelength. While the AB structure at frame 7 remains relatively intact, due to the strong seeding the inter and intra-pulse distortions grow as the AB advances along z, leading to the elimination of the traces of the AB entirely.

Remarkably, despite the local structural deformation, the periodicity of the AB in both the time and phase fields remains robustly intact. As shown in Fig. 6(c) and Fig. 6(d), the transverse periodicity is consistently sustained across all frames, this means that this is true even for infinitesimally small steps along z. This preserved periodicity is crucial for the formation of consistent patterns and plays a fundamental role in the evolution of the AB. Furthermore, it serves as a key mechanism underlying the emergence of complex patterns commonly observed in natural systems.

[1] J. M. Soto-Crespo, N. Devine, and N. Akhmediev. Integrable turbulence and rogue waves: Breathers or solitons? Physical Review Letters, 116(10):103901, 2016.

[2] Stéphane Randoux, Pierre Suret, and Gennady El. Inverse scattering transform analysis of rogue waves using local periodization procedure. Scientific reports, 6(1):1–11, 2016.

[3] E. A. Kuznetsov. Solitons in a parametrically unstable plasma. Dokl. Akad. Nauk SSSR, pages 575–577, (1977).
